# Trial‐by‐trial co‐variation of pre‐stimulus EEG alpha power and visuospatial bias reflects a mixture of stochastic and deterministic effects

**DOI:** 10.1111/ejn.13688

**Published:** 2017-09-28

**Authors:** Christopher S. Y. Benwell, Christian Keitel, Monika Harvey, Joachim Gross, Gregor Thut

**Affiliations:** ^1^ Centre for Cognitive Neuroimaging Institute of Neuroscience and Psychology University of Glasgow 58 Hillhead Street Glasgow G12 8QB UK; ^2^ School of Psychology University of Glasgow Glasgow UK

**Keywords:** attention, EEG, jackknife, line bisection, oscillations

## Abstract

Human perception of perithreshold stimuli critically depends on oscillatory EEG activity prior to stimulus onset. However, it remains unclear exactly which aspects of perception are shaped by this pre‐stimulus activity and what role stochastic (trial‐by‐trial) variability plays in driving these relationships. We employed a novel jackknife approach to link single‐trial variability in oscillatory activity to psychometric measures from a task that requires judgement of the relative length of two line segments (the landmark task). The results provide evidence that pre‐stimulus alpha fluctuations influence perceptual bias. Importantly, a mediation analysis showed that this relationship is partially driven by long‐term (deterministic) alpha changes over time, highlighting the need to account for sources of trial‐by‐trial variability when interpreting EEG predictors of perception. These results provide fundamental insight into the nature of the effects of ongoing oscillatory activity on perception. The jackknife approach we implemented may serve to identify and investigate neural signatures of perceptual relevance in more detail.

## Introduction

Understanding how sensory information is sampled and encoded by the brain represents an ongoing challenge in neuroscience. Progress has been made with regard to the macroscopic processes underlying perception through the development of functional‐anatomical and network dynamic models, based on lesion studies and cognitive neuroimaging (see Petersen & Posner, 2012; Corbetta & Shulman, 2011 and Vossel *et al*., 2014 for reviews), as well as electrophysiological studies in animals and humans (Engel *et al*., 2001; Jensen & Mazaheri, 2010; Thut *et al*., 2012; Keitel & Gross, 2016). Within the latter field, many studies have employed EEG/MEG to examine how specific neural states, as indexed by oscillatory activity prior to stimulus onset, predict the perceptual fate of an upcoming stimulus. These studies have identified pre‐stimulus phase (Busch *et al*., 2009; Mathewson *et al*., 2009, 2011; Busch & VanRullen, 2010; Fiebelkorn *et al*., 2011, 2013; VanRullen *et al*., 2011; Milton & Pleydell‐Pearce, 2016) and/or power (Thut *et al*., 2006, 2012; Wyart & Tallon‐Baudry, 2009; Klimesch, 2012; Kelly & O'Connell, 2013; Capilla *et al*., 2014) in specific frequency bands as covariates of perceptual outcome. For example, pre‐stimulus oscillatory power in the alpha band (~8:14 Hz) over occipito‐parietal sites has been shown to be inversely related to the likelihood of detecting a perithreshold visual stimulus (Ergenoglu *et al*., 2004; Van Dijk *et al*., 2008; Busch *et al*., 2009). Additionally, the relative lateralization of alpha power between left and right posterior brain regions has been found to predict visual field reaction time (RT) asymmetries in lateralized detection tasks, that is to predict spatial bias (Thut *et al*., 2006; Kelly *et al*., 2009; Newman *et al*., 2013, 2016). While early research mainly revealed the pre‐stimulus predictors of binary decisions in threshold detection tasks (i.e. percept vs. no percept), recent EEG studies have employed psychophysical modelling techniques (Chaumon & Busch, 2014; Limbach & Corballis, 2016; Iemi *et al*., 2017; Samaha *et al*., 2017). Allowing for a more detailed interrogation of the functional roles of pre‐stimulus activity in perception, these studies provide emerging evidence that pre‐stimulus alpha activity may primarily bias perceptual decisions rather than improving visual sensitivity in tasks with perithreshold stimuli.

Here, we sought to investigate whether bias and/or sensitivity are influenced by pre‐stimulus oscillatory activity during performance of a psychophysical task with suprathreshold stimuli. We employed a task that requires the judgement of the relative length of two segments of a horizontally presented line; the landmark task (Milner *et al*., 1992; McCourt & Olafson, 1997). While originally developed as a diagnostic tool in hemispatial neglect (Harvey *et al*., 1995), the task also provides a sensitive measure of the pseudoneglect phenomenon. Pseudoneglect represents a perceptual bias towards the left side of space and/or objects that is found in the majority of neurologically normal young adults (Bowers & Heilman, 1980; Fink *et al*., 2000; Jewell & McCourt, 2000; Foxe *et al*., 2003; Benwell *et al*., 2013a,2013b, 2014a,2014b, 2015). Importantly for our study, the landmark task measures not only visuospatial bias but also visual (size) discrimination sensitivity through common psychometrics.

An intriguing aspect of landmark task performance is that visuospatial bias often changes over the course of the experimental session (time‐on‐task effect: Manly *et al*., 2005; Dufour *et al*., 2007; Benwell *et al*., 2013a,2013b; Veniero *et al*., 2017), with the initial group‐level leftward bias repeatedly being found to shift rightward over time. Likewise, visual discrimination sensitivity may change progressively over time. This allowed us to examine more closely an open question on the relationship(s) between pre‐stimulus oscillations and perception; namely to what extent do they follow a stochastic pattern across trials (i.e. are driven by ‘spontaneous’ trial‐by‐trial variability). Alternatively, they may rather be explained by deterministic endogenous or exogenous sources of variance such as trial order, fatigue, adaptation or practice effects (Monto *et al*., 2008; de Lange *et al*., 2013; Newman *et al*., 2013; Bompas *et al*., 2015). Slow drifts in EEG characteristics such as increase/decrease in spectral power in the order of seconds, minutes and hours provide candidate mechanisms for non‐stationarity in psychophysical performance over time (Makeig & Jung, 1995; Fründ *et al*., 2011; Doll *et al*., 2015) and may therefore contribute to the frequently reported covariance between pre‐stimulus activity and performance.

Hence, the aims of the current study were twofold: (1) to identify within the same participants pre‐ (and post‐) stimulus oscillatory covariates of psychometric measures of spatial bias and discrimination sensitivity derived from landmark task performance and (2) to assess whether any identified link is determined by time‐on‐task (i.e. is deterministic) or rather originates from spontaneous trial‐by‐trial variability (i.e. is stochastic). To relate ongoing EEG activity to the psychometric measures not defined at the single‐trial level (aim 1) and to assess the potential deterministic role of time‐on‐task over trials (aim 2), we implemented a single‐trial analysis procedure (based on jackknife‐estimated correlations: Quenouille, 1949; Tukey, 1958; Parr, 1985; Stahl & Gibbons, 2004; Richter *et al*., 2015), in combination with a mediation analysis (Kenny *et al*., 2003; Wager *et al*., 2008).

## Materials and methods

### Participants

Twenty individuals volunteered to participate in this study and were financially compensated for their time. One participant was excluded from subsequent analysis due to inconsistent use of response keys which precluded psychophysical analysis of the data. Hence, analyses were carried out on the data of 19 individuals (seven males, 12 females, mean age: 24 years, min: 17, max: 33). All participants were right‐handed with normal or corrected‐to‐normal vision and no history of neurological disorder. Written informed consent was obtained from each participant, and the study was approved by the local ethics committee. The experimental sessions were carried out within the Institute of Neuroscience and Psychology at the University of Glasgow.

### Instrumentation and stimuli

**Figure 1:**
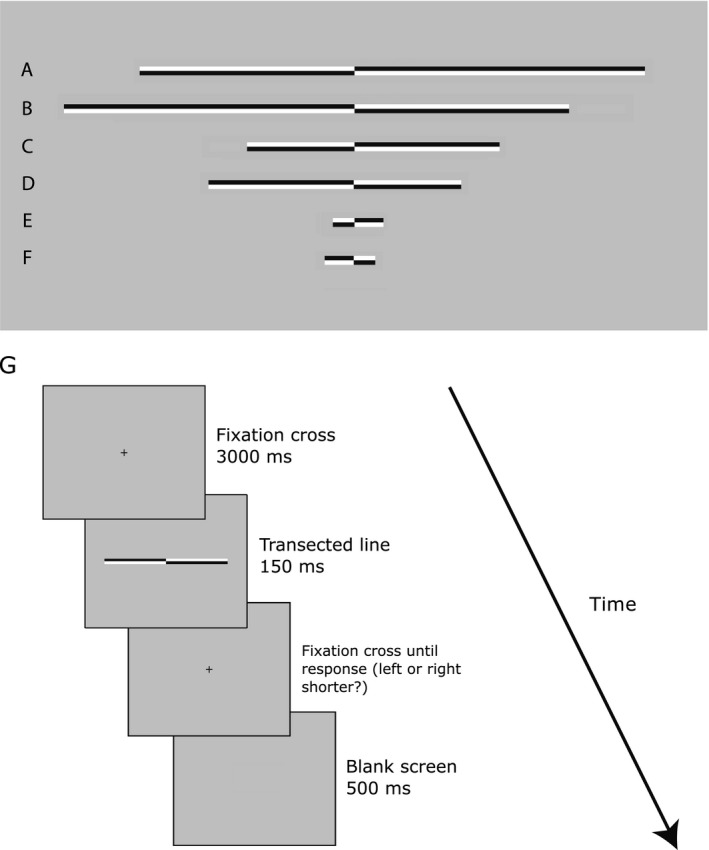
Stimuli and trial procedure. The task involved the judgement of the relative length of two segments of a horizontally presented line (the landmark task). (A‐F) Line stimuli used in the experiment. Lines A and B represent long lines, lines C and D represent medium lines and lines E and F represent short lines. Lines were transected at 1 of 13 locations ranging from ±7.5% of absolute line length relative to the veridical mid‐point. Lines A, C and E are transected to the left of veridical centre whereas lines B, D and F are transected to the right of veridical centre. (G) A schematic representation of the trial procedure. Following presentation of a fixation cross for 3000 ms, a transected line was presented for 150 ms before reappearance of the fixation cross on the screen until the participant responded by pressing either the left or right ‘shorter’ response key.

The task was a computerized version of the landmark task (Milner *et al*., 1992; McCourt & Olafson, 1997) in which participants were asked to estimate which of two segments of a pre‐bisected horizontal line was shortest. The stimuli were presented using the E‐Prime software package (Schneider *et al*., 2002) on a CRT monitor with a 1280 × 1024 pixel resolution and 85 Hz refresh rate. The stimuli were identical to those employed in Benwell *et al*. (2014b). Figure 1 (A:F) shows examples of line stimuli used in the experiment. Three different line lengths were presented. ‘Long’ lines (Fig. 1A,B) measured 24.3 cm in length (subtending 19.67° visual angle (VA) at a viewing distance of 70 cm)  × 0.5 cm in height (0.4° VA). ‘Medium’ lines (Fig. 1C,D) measured 12.15 cm (9.92° VA) × 0.5 cm (0.4° VA) and ‘short’ lines (Fig. 1E,F) measured 2.43 cm (1.98° VA) × 0.5 cm (0.4° VA). Line length was manipulated in analogy with previous studies (Jewell & McCourt, 2000; Benwell *et al*., 2013a, 2014a,2014b) but potential effects of line length were not analysed here in terms of associated EEG features (as they are planned to be the subject of another report).

All three line lengths were transected at one of 13 locations with a range of ±7.5% of absolute line length relative to the veridical mid‐point (distance between transector locations = 1.25%). In long vs. medium vs. short lines, this represented a range of ±1.48° vs. ±0.74° vs. ±0.15° VA (with a distance between transector locations of 0.25° vs. 0.12° vs. 0.02° VA). All lines were displayed with the transector location centred on the vertical midline of the display (i.e. aligned to a central fixation cross which preceded the presentation of the lines), thereby preventing use of the position of the fixation cross relative to the transection mark as a reference point for judgements.

### Procedure

Participants were seated 70 cm from the display monitor with their mid‐sagittal plane aligned to the centre of the screen. Viewing distance was kept constant throughout the experiment using a chin rest. During landmark task performance, each trial began with presentation of a black fixation cross (subtending 0.4° × 0.4° VA) which remained on the screen for 3 seconds (s) followed by presentation of the transected line (0.15 s). Following the disappearance of the line, the fixation cross remained on the screen until the participant indicated which end of the line had appeared shortest to them by pressing either the left (‘v’) or right (‘b’) response key with their dominant right‐hand (right index and middle finger, respectively) (see Fig. 1G for a schematic representation of the trial procedure). Participants were instructed to maintain their gaze on the fixation cross throughout each trial. The subsequent trial began 0.5 s after the response was made (a blank grey screen was displayed throughout this period). Hence, trials lasted approximately 4–5 s. Trial type (length and location of transector in line) was selected at random. Each participant completed 234 trials at each line length (Overall = 702 trials, 18 judgements at each of the transector locations per line length) split into nine blocks (lasting approximately 6–7 min each). Participants were allowed to take a short break between blocks. The entire experiment lasted approximately 1 h.

### Behavioural analysis

The behavioural measures of interest were the point of subjective equality (PSE) and curve width derived from psychometric functions fit to the landmark task responses. The dependent measure was the proportion of trials on which participants indicated that the transector had appeared closer to the left end of the line (i.e. left segment shorter responses). The proportion left response was calculated for all transector locations and a generalized linear model with a logit link function was used to fit psychometric curves (using the Matlab ‘glmfit’ function (Mathworks, USA)). The logit link function is described by the following:

$$B*X=\log\left(\frac{\mu }{1-\mu }\right)$$

where **μ** is the mean ‘left shorter’ response, **X** are the transector locations and **B** is a two element coefficient vector (*B*_1_, *B*_2_). These coefficients determine a logistic function defined as:

$$\text{left shorter response}=\frac{1}{1+\exp\left(-\left(B_{1}*X+\;B_{2}\right)\right)}$$

The PSE is the value on the x‐axis (transector locations) where the participant is at 50% performance and was calculated as follows:

$$\text{PSE}=\frac{-B_{1}}{B_{2}}$$

The PSE represents an objective measure of perceived line mid‐point. Values below zero (‐) index a leftward bias, indicating that the participant tends to overestimate the left segment of the line and only perceives the two ends of the line to be equal when the right segment is actually objectively longer. Positive values (+) indicate an opposite rightward bias. The curve width (steepness of the fitted curve) was calculated from the difference along the x‐axis between 75% and 25% performance on the y‐axis:

$$\text{Curve width}=\;\frac{{\text{logit}}_{25}-\;{\text{logit}}_{75}}{B_{2}}$$

where logit_25_ and logit_75_ correspond to the logit transforms of 0.25 and 0.75, which are the values on the y‐axis corresponding to 25% and 75% left shorter responses, respectively. The curve width indexes discrimination sensitivity for the task, providing a measure of the precision of participants’ relative length judgements.

Initially, inferential statistics were performed on the PSE and curve width values from individually fitted psychometric functions to ascertain known neurotypical performance patterns in this task (pseudoneglect) and the influence of time‐on‐task (changes in spatial bias over the course of the experimental session) with the data split into three equally sized sequential bins (comprised of three ‘blocks’ each in order to obtain a sufficient number of trials for stable psychometric function fitting) spanning the course of the experimental session. Subsequent to this and central to our objective, the psychometric function parameters described were calculated within a jackknife single‐trial analysis approach (see below) in order to investigate task relevant oscillatory EEG signatures at the single‐trial level.

### Electrophysiological measures

Continuous electroencephalogram (EEG) recording was acquired with a BrainAmp MR Plus unit (Brain Products GmbH, Munich, Germany) at a sampling rate of 1000 Hz through 60 scalp electrodes and four ocular electrodes (horizontal and vertical bipolar montage). Impedance was kept below 10 KΩ. Pre‐processing steps were performed using a combination of custom scripts incorporating EEGLAB (Delorme & Makeig, 2004) and FieldTrip (Oostenveld *et al*., 2011) functions in Matlab (Mathworks, USA). Offline, continuous data were filtered for power line noise using a notch filter centred at 50 Hz. Additional low (100 Hz) and high‐pass (0.1 Hz) filters were applied using a zero‐phase second‐order Butterworth filter. The data were then divided into epochs spanning −2.5:1.5 seconds (s) relative to stimulus onset on each trial. Subsequently, excessively noisy electrodes were removed without interpolation, the data were re‐referenced to the average reference (excluding ocular channels) and trials with abnormal activity were rejected using a semi‐automated artefact detection procedure which highlighted trials with potential artefacts based on a) extreme amplitudes (threshold of ± 75 μV), b) joint probability of the recorded activity across electrodes at each time point (probability threshold limit of 3.5 (single‐channel limit) and 3 (global limit) standard deviations (std. dev), respectively (pop_jointprob; Delorme & Makeig, 2004)) and c) kurtosis (local limit of 5 SD and global limit of 3 SD (pop_rejkurt; Delorme & Makeig, 2004)). Consequently, the mean number of trials entered for further analysis was 595 (84.7%, minimum = 491 (70%), maximum = 677 (96.4%) across participants). An independent component analysis (ICA) was then run using the runica EEGLAB function (Delorme & Makeig, 2004) and components corresponding to blinks, eye movements and muscle artefacts were removed. Missing channels were then interpolated using a spherical spline method and the trial mean was removed from all electrodes (whole‐epoch baseline correction). Fourier‐based spectro‐temporal decomposition of the artefact‐removed single‐trial data was performed using the ft_freqanalysis function (Oostenveld *et al*., 2011) with the ‘mtmconvol’ option. This implementation yields a complex‐valued time‐frequency plane for each trial. A temporal resolution was maintained by decomposing overlapping 0.5 s segments of trial time series, consecutively shifted forward in time by 0.02 s. Data segments were multiplied with a Hanning taper and then zero‐padded to a length of 2 s to achieve a frequency resolution of 0.5 Hz across the range of 1:30 Hz. The data were then re‐epoched from −2:1 s relative to stimulus onset to exclude artefacts arising at the edges of transformed time series.

We sought to investigate spectral EEG signatures of distinct measures of visuospatial attention performance as indexed by landmark task psychometric function parameter estimates (i.e. PSE and curve width). Our analysis focussed on oscillatory power. Single‐trial power was obtained for all time‐frequency points as follows:

$$\text{abs power}\;\left(t,f\right)={\left|F\left(t,f\right)\right|}^{2}$$

where *F* is the complex Fourier coefficient corresponding to time window *t* and frequency *f*. The absolute power values were additionally normalized to the average power of each frequency band across the whole‐epoch using a decibel (dB) transformation:

$$\text{dB power}\left(t,f\right)=10\log_{10}\left(\frac{\text{abs power}(t,f)}{\text{mean abs power}(f)}\right)$$

Single‐trial analyses were conducted using a jackknife procedure as described below.

### Joint EEG and psychophysics jackknife analysis

Recently, single‐trial analyses linking EEG activity and metrics of interest defined at the single‐trial level (i.e. decision outcome, reaction time, stimulus properties) have been used to investigate the relationship between brain activity and behaviour/perception (see for example Busch *et al*., 2009; Ratcliff *et al*., 2009; Cohen & Cavanagh, 2011; Pernet *et al*., 2011; Schyns *et al*., 2011; Cohen & Donner, 2013; Gross, 2014) unobservable in traditional analyses where trial‐by‐trial variance is eradicated through averaging of data at the single‐subject level. Typically, single‐trial neural activity is correlated with single‐trial behavioural measurements such as reaction time or accuracy (Kelly & O'Connell, 2013; Newman *et al*., 2016). However, psychometric function parameter estimation requires the input of behavioural responses across multiple trials and hence cannot be defined based on single‐trial observations. Richter *et al*. (2015) recently proposed a jackknife approach to estimating single‐trial correlations between EEG metrics that are not necessarily defined at the single‐trial level, for instance inter‐trial phase coherence. The jackknife is a leave‐one‐out resampling technique in which a chosen parameter of a dataset is calculated by systematically leaving out each single observation and calculating the parameter over all remaining observations (Quenouille, 1949; Tukey, 1958; Parr, 1985). Hence, given a sample size of ***N***, each jackknife estimate of the parameter is calculated from ***N‐1*** data points of the sample thereby resulting in ***N*** ‘jackknife replications’ of the estimate. The resulting estimates fluctuate somewhat across the jackknife replications, capturing variance in the original observations of the measure of interest (Richter *et al*., 2015). The jackknife replications can then be correlated with any other measure which by itself may or may not be defined at the level of single observations, providing an estimate of covariance between the two measures across the trials of an experiment for example. Richter *et al*. (2015) convincingly demonstrated the superiority of jackknife correlations over commonly employed sorting‐and‐binning approaches in which trials are binned according to the value of one variable and the metric of interest is calculated for the other variable across all trials in each bin. We sought to identify spectral EEG signatures of distinct measures of visuospatial attention performance as indexed by landmark task psychometric function parameter estimates. Specifically, our EEG measures of interest were the pre‐ and post‐stimulus power and our psychophysical measures of interest were the PSE and curve width. Each separate analysis consisted of two levels, an initial single‐trial analysis within subjects, followed by a group‐level analysis, both of which are outlined below.

### EEG power vs. psychometric measures

#### Within‐participant analysis

The first‐level (within participant) analysis consisted of a linear regression performed between the jackknife replications of the mean EEG power and the corresponding psychophysical measure of interest of the samejackknife sample. The leave‐one‐trial‐out jackknife replications (JKR) for each measure of interest were calculated as follows:

$${\text{JKR}}_{i}=S\left(k_{1},k_{2},\cdots ,k_{i}-1,k_{i}+1,\cdots ,k_{n}\right)$$

where ***S*** is the measure of interest (i.e. mean EEG power, PSE, curve width) calculated over the trials ***k***. ***k***_***i***_ is a single trial for which the value contributing to the calculation of the measure of interest has been left out. Hence, JKR_*i*_ (the jackknife replication) captures the small change in the measure of interest ***S*** without trial ***k***_***i***_. This process is repeated ***n*** times as each of the trials ***k*** are systematically left out resulting in ***n*** jackknife replication values. Note that this procedure results in compression and inversion of the ***k*** distribution, but the variance of the resulting **JKR** distribution represents a precise transform of the variance of the ***k*** distribution. The compression arises from the range of **JKR** values being smaller in absolute terms than the range of ***k*** values because **JKR** values represent small changes in ***S*** induced by leaving one trial out. The inversion arises because a relatively ‘low’ value of **JKR**_***i***_ indicates a relatively ‘high’ value of ***k***_**i**_ (i.e. if a high value is removed from a distribution then the mean will be reduced) but as the inversion occurs for both variables the true direction of the relationship is preserved. Hence, neither the compression nor inversion of the distribution precludes the subsequent analyses explained below.

Employing a linear regression approach, the single‐trial data were modelled as follows:

$$J_{\mathrm{Psy}}\left(t,f\right)=\;\beta_{1}\left(t,f\right)+\;\beta_{2}\left(t,f\right)*J_{\mathrm{EEG}}\left(t,f\right)$$

***JPsy*** represents the jackknife replications of the psychometric function measure of interest (PSE or curve width) at a given time‐frequency point (***t,f***). **JEEG** represents the jackknife replications of the EEG power at a given time‐frequency point (***t,f***). The regression coefficient **β2** represents the slope of the fitted regression line and indicates the direction and strength of the relationship between the two variables (**β1** represents the intercept and was not further analysed). This regression analysis was performed separately for all electrodes and time‐frequency points in each participant, returning a three‐dimensional matrix (electrodes, frequencies, time points) of **β2** values which were entered into the second‐level group analysis described below.

#### Group‐level analysis

We sought to identify spectral characteristics of the EEG which linearly co‐varied with the psychophysical measures. If at a given data point (electrode/frequency/time), the value of the EEG power systematically co‐varies linearly with the psychometric parameter (PSE or curve width), then regression slopes should show a consistent directionality across subjects. Alternatively, if there is no systematic linear relationship between the values of the EEG and psychometric parameters, then regression slopes across subjects should be random (centred around 0). Hence, for each EEG/psychophysics relationship we performed one‐sample *t*‐tests (test against 0) on the β2 (regression slope) values across participants at all data points (i.e. all electrodes, frequencies, time points). To control the familywise error rate (FWER) across the large number of comparisons, cluster‐based permutation testing (Maris & Oostenveld, 2007) was employed.

In line with Maris & Oostenveld (2007), the calculation of the test statistic involved the following: based on the initial one‐sample *t*‐tests, all *t*‐values above a threshold corresponding to an uncorrected *P*‐value of 0.05 were formed into clusters by grouping together adjacent significant time‐frequency points and electrodes. This step was performed separately for samples with positive and negative *t*‐values (two‐tailed test). Note that for a significant sample to be included in a cluster it was required to have at least one adjacent neighbouring significant sample**.** The spatial neighbourhood of each electrode was defined as all electrodes within approximately 5 cm, resulting in a mean of 6.3 (min = 3, max = 8) and median of 7 neighbours per electrode. The *t*‐values within each cluster were then summed to produce a cluster‐level t‐score (cluster statistic). Subsequently, this procedure was repeated across 2000 permutations to create surrogate data using ‘ft_statistics_montecarlo’ (Oostenveld *et al*., 2011). On each iteration, this function effectively switched the sign of the regression slope for a random subset of the participants. A one‐sample *t*‐test of regression slopes against zero was then performed at each data point. After clustering *t*‐values across data points, the most extreme cluster‐level t‐score was retrieved to build a data‐driven null hypothesis distribution. The location of the original real cluster‐level t‐scores within this null hypothesis distribution indicates how probable such an observation would be if the null hypothesis were true (no systematic difference from 0 in β2 across participants). Hence, if a given negative/positive cluster had a cluster‐level t‐score lower/higher than 97.5% of the respective null distribution t‐scores, then this was considered a significant effect (5% alpha level).

In order to compare the results of the jackknife analysis to a more traditional approach, we also performed a ‘high’ vs. ‘low’ power trial binning analysis which is reported in the supplementary materials.

### Mediation analysis

**Figure 2:**
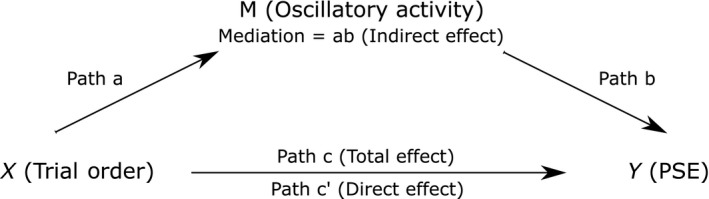
Mediation hypothesis. Diagram of our mediational hypothesis that the effect of trial order on spatial bias may be mediated by oscillatory power.

We hypothesize that a potential explanatory third variable with regard to any observed relationship between EEG activity and subjective mid‐point estimates (PSE) is time‐on‐task (see Manly *et al*., 2005; Benwell *et al*., 2013a; Newman *et al*., 2013). Mediation analysis tests whether a covariance between two variables (***X*** and ***Y***) can be explained by a third variable (***M***) (Baron & Kenny, 1986; Kenny *et al*., 2003). Hence, to test whether trial order may mediate any relationship between EEG activity and PSE, multilevel mixed‐effects mediation analyses were carried out using the Mediation Toolbox (http://wagerlab.colorado.edu/tools (Wager *et al*., 2008; Woo *et al*., 2015)). For each analysis, the first level involved calculation of participant‐level path estimates using linear regression. To test the involvement of time‐on‐task (indexed by trial order), the ***X*** variable was the vector of single‐trial jackknife replications of the trial indices (1:***N*** trials), the ***Y*** variable was the vector of single‐trial jackknife replications of the PSE and the ***M*** variable was the vector of single‐trial jackknife replications of the EEG measure of interest (oscillatory power) (see also Fig. 2). Trial order was assigned as the ***X*** (causal) variable and oscillatory power as the mediator because ***M*** must be causally located between ***X*** and ***Y*** (i.e. the EEG measure cannot be employed as the causal variable as it is nonsensical to posit that it may cause the trial order). A significant mediator is one whose inclusion as an intermediate variable in a path model of the effects of ***X*** on ***Y*** (see Fig. 2 for paths) significantly changes the slope of the ***X***‐***Y*** relationship. Hence, path ***a*** represents the estimated linear change in EEG power per unit change in trial order. Path ***b*** is the slope of the EEG‐PSE relationship controlling for trial order, and paths ***c*** and ***c’*** represent the total trial order‐PSE effect and the direct trial order‐PSE effect, respectively. The indirect trial order‐PSE effect (***ab***) equals the reduction of the trial order‐PSE effect when EEG is included in the model and so quantifies the amount of mediation:

$$c=c^{\prime }+ab$$

If trial order no longer affects PSE after EEG power has been controlled for (path ***c’*** = 0) then this represents a case of complete mediation. If the effect of trial order on PSE is reduced by controlling for EEG power but remains significant (path ***c’*** > 0 but < ***c***) then this represents a case of partial mediation. Within a two‐level model in which all three variables are random effects (allowed to vary across participants), an additional term (σ*ab*) can also explain a reduction in the group‐level trial order‐PSE effect when EEG power is included in the model:

$$c=c^{\prime }+ab+\sigma ab$$

where **σ** ***ab*** is the population covariance of paths *a* (trial order‐ EEG power) and *b* (EEG power ‐PSE). A positive value of **σ** ***ab*** implies a positive correlation between paths *a* and *b* across participants whereas a negative value indicates a negative correlation. To clarify the difference between ***ab*** and **σ** ***ab***,***ab*** represents the degree to which the X‐Y relationship is reduced within a given participant when the proposed mediator is controlled for, whereas ***σab*** is the correlation between paths ***a*** and ***b*** across participants (Kenny *et al*., 2003). In our case, ***ab*** represents the reduction in the trial order‐PSE relationship when changes in oscillatory power over trials are controlled for and **σ** ***ab*** represents the correlation between the strength of the trial order‐alpha power relationship and the strength of the alpha power‐PSE relationship across participants. Both of these measures can signal the existence of mediation because there are two ways in which the direct effect (***c’***) might be less than the total effect (***c***) in a multilevel mediation model. First, there is reduction if ***ab*** is systematically nonzero across participants, or second, if the between‐subject correlation between paths ***a*** and ***b*** (**σ** ***ab***) is nonzero, or a combination of both. Note that ***ab*** might equal zero, but **c’** can still be less than **c** because **σ** ***ab*** can be nonzero. Therefore, for each EEG power/PSE effect, we tested for systematic directionality of the ***ab*** (mediation) effect (i.e. are ***ab*** slopes across participants significantly different from zero?) at all data points (electrodes/frequencies/ times). Cluster‐based permutation tests were again employed for multiple comparison correction. Further to any detected mediation (***ab***) effects, we also correlated paths ***a*** (trial order‐ EEG power) and ***b*** (EEG power ‐PSE) across participants to test whether **σ** ***ab*** was also nonzero.

### Regions of interest (ROI) analysis on lateralization index of power between hemispheres

Based on previous studies investigating the relationship between the relative lateralization of pre‐stimulus posterior alpha power between left and right hemispheric ROIs and the horizontal distribution of spatial attention across the visual fields (Thut *et al*., 2006; Newman *et al*., 2013, 2016; Loughnane *et al*., 2015; Slagter *et al*., 2016), and to facilitate comparison to this literature, we performed an additional analysis of the asymmetry in alpha activity based on ROIs to investigate whether this measure also predicts subjective mid‐point estimates on the landmark task both between and within participants. In order to try to improve topographic localization of alpha power separately for the left and right ROI, this analysis was performed on data calculated with a surface Laplacian (Perrin *et al*., 1989; Cohen, 2014). The surface Laplacian, a term which is interchangeable with current source density (CSD), is a spatial filter which yields topographies with reduced volume conductance (Kayser & Tenke, 2015). This allows for improved topographic localization of underlying cortical sources of rhythmic brain activity (Keitel *et al*., 2013, 2017; see also Tenke & Kayser, 2015). The surface Laplacian was calculated prior to time‐frequency transformation.

Single‐trial estimates of posterior alpha lateralization were calculated as follows:

$$\frac{\text{LI}=\text{alpha}\;(\text{right ROI})-\text{alpha}(\text{left ROI})}{(\text{alpha}\;(\text{right ROI})+\text{alpha}(\text{left ROI}))/2}$$

The lateralization index (LI) is negative when alpha power is stronger over the left than the right hemisphere and positive when alpha is stronger over the right than the left hemisphere. Mean alpha power was calculated from 8 to 12 Hz separately for both pre‐stimulus (−2: −0.5 s) and post‐stimulus time periods (0.02:0.8 s, corresponding to stimulus‐induced alpha desynchronization). In line with relevant previous studies investigating the effect of pre‐stimulus alpha lateralization on attention (Thut *et al*., 2006; Newman *et al*., 2013, 2016; Loughnane *et al*., 2015), the right hemisphere (RH) ROI covered electrodes P4, P6, P8, PO4, PO8 and O2 and the left hemisphere (LH) ROI covered electrodes P3, P5, P7, PO3, PO7 and O1. A 2 (hemisphere: left, right) x 2 (time: first block, final block) anova was performed to test for overall hemispheric asymmetry in alpha power and whether this asymmetry changed over the course of the experiment. Additionally, a between‐subject correlation analysis was performed to test for a relationship between mean alpha LI values and spatial bias at all time points across participants and the jackknife approach was used to test for a single‐trial relationship between alpha LI values and spatial bias within participants. For the between‐participant correlation analysis, a robust measure of statistical association (Shepherd's pi) was implemented which is equivalent to Spearman's rho after outlier removal (Schwarzkopf *et al*., 2012).

### EEG phase correlates of psychophysical measures

Finally, in addition to spectral power, we also investigated the link between pre‐stimulus phase and psychophysical measures. This analysis was motivated by previous research showing a link between the alignment of oscillatory phase and perceptual outcome (Busch *et al*., 2009; Busch & VanRullen, 2010; VanRullen *et al*., 2011; Fiebelkorn *et al*., 2011, 2013; Mathewson *et al*., 2009, 2011; Milton & Pleydell‐Pearce, 2016; however see Chaumon & Busch, 2014 and van Diepen *et al*., 2015 for negative findings).

Inter‐trial phase coherence (ITPC) was calculated as follows:

$$\text{ITPC}\left(t,f\right)=|\frac{1}{n}\sum_{k=1}^{n}\frac{F_{k}\left(t,f\right)}{|F_{k}\left(t,f\right)|}|$$

where *F* is the complex Fourier coefficient corresponding to time window *t* and frequency *f*,* n* is the number of trials and *k* is the individual trial index. Single‐trial jackknife replications of the ITPC values were calculated and entered into the same regression analysis, that is single‐trial jackknife EEG vs. PSE and curve width replications, respectively, as for the power analysis.

The ITPC analysis tests whether pre‐stimulus phase‐locking linearly changes from one perceptual outcome to another (i.e. ‘left shorter’ to ‘right shorter’ (PSE) or from ‘low discrimination sensitivity’ to ‘high discrimination sensitivity’ (curve width)). However, a possible scenario that could be missed by such an analysis is that both outcomes could be phase‐locked to opposing phase angles (see VanRullen, 2016). Hence, in addition to correlating PSE and curve width with ITPC, we also tested whether different perceptual outcomes for each measure show significant differences in preferred phase angle. To do so, we employed the circular Watson–Williams test (Zar, 1999; Berens, 2009). For the spatial bias measure, we simply coded each trial in terms of ‘left shorter’ or ‘right shorter’ responses. For the discrimination sensitivity measure, we used the vector of jackknife replications to split the data into above and below median relative discrimination performance. In order to test for statistical significance, a *P*‐value was obtained for each participant at each electrode‐time‐frequency point separately and the resulting single‐participant *P*‐values were combined to test for significance at the group‐level using a method described by Stouffer *et al*. (1949) (see VanRullen, 2016 for further details). To control for multiple comparisons, we employed a parametric FDR‐correction (Benjamini & Yekutieli, 2001). The entire analysis was performed separately for spatial bias and visual discrimination sensitivity, respectively.

## Results

### Behavioural results

**Figure 3:**
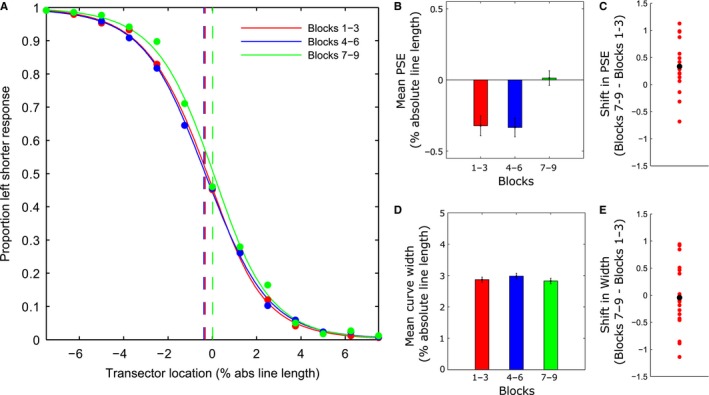
Behavioural Results. Participants displayed a group‐level leftward bias (pseudoneglect) at baseline which systematically shifted rightwards over the course of the experimental session (time‐on‐task effect). (A) Group‐averaged psychometric functions for each third of the experiment (collapsed across line lengths). For each time bin, filled circles plot the mean proportion of ‘left shorter’ responses as a function of transector location. Red (Blocks 1–3), blue (Blocks 4–6) and green (Blocks 7–9) vertical dashed lines indicate the group‐averaged point‐of‐subjective equality's (PSEs). (B) Group‐averaged PSE values (±1 within‐subject standard error (SE) (Cosineau, 2005)) obtained from psychometric functions fit to the individual participants’ data for each third of the experiment. Negative values represent a leftward bias (tendency to overestimate/underestimate length of the left/right line segment, respectively). (C) Differences in PSE between the final blocks (7–9) and the initial blocks (1–3). Positive values indicate a rightward shift. Red dots indicate individual participant differences, highlighting the consistency of the rightward shift in subjective mid‐point over time. The black dot indicates the group mean difference. (D) Group‐averaged psychometric function curve width values (±1 within‐subject SE) for each third of the experiment. (E) Differences in curve width between the final blocks (7–9) and the initial blocks (1–3). Red dots indicate individual participant differences, highlighting the inconsistency of changes in curve width over time (i.e. no systematic change). The black dot indicates the group mean difference.

The landmark task involved the judgement of the relative length of two segments of a horizontally presented line that could be transected in each trial at one of several different positions (see Fig. 1). The behavioural measures of interest were spatial bias (indexed by the PSE) and discrimination sensitivity (indexed by psychometric function curve width). These were derived from psychometric functions (PFs) fitted to the behavioural responses. We observed the neurotypical pattern of line bisection performance, with baseline pseudoneglect (leftward bias) and a rightward shift over time as shown in many previous studies, indicating that participants performed the task as expected. Figure 3A shows group‐averaged psychometric functions for each third of the experiment. Group‐level PSE's for both the first and second third of the experiment were located significantly to the left of the veridical centre (95% confidence intervals (CIs) do not include 0) indicating that, on average, participants perceived the two segments to be equally long for left bisected lines (i.e. when the left segment was in reality shorter than the right). This leftward bias was attenuated and not significantly different to veridical centre during the final third of the experiment (see vertical dashed lines in Fig. 3A, corresponding to 50% left/right responses: PSE Block 1 (95% CI)  = −0.321 (−0.453: −0.171)); PSE Block 2 = −0.328 (−0.463: −0.175); PSE Block 3 = 0.016 (−0.133: 0.186)).

Figure 3B shows the mean PSE's (% of absolute line length relative to veridical centre) from individually fitted PFs for each third of the experiment. A repeated‐measures anova on the PSE values with time‐on‐task as a within‐subject factor (three levels) revealed a significant main effect (*F*_2,36_ = 6.486, *P *= 0.004, $\eta_{p}^{2}$ = 0.265). In line with previous findings (Manly *et al*., 2005; Benwell *et al*., 2013a,2013b), the PSE shifted rightwards over the course of the experimental session. Pairwise comparisons employed to analyse the effect of time‐on‐task revealed no statistically significant difference in PSE between the first and second bins (*t*_18_ = −0.095, *P* = 0.925, Cohen's *d *=* *0.022) but a statistically significant rightward shift in PSE between the second and third bins (*t*_18_ = 3.636, *P* = 0.002, Cohen's *d *=* *0.933) and the first and third bins (*t*_18_ = 3.226, *P* = 0.005, Cohen's *d *=* *0.763). Additionally, a linear contrast analysis across the three time bins revealed a significant rightward linear shift in bias over the course of the session (*F*_1,18_ = 9.991, *P* = 0.005, $\eta_{p}^{2}$ = 0.366). Figure 3C plots the difference in PSE between the final blocks (7–9) and the initial blocks (1–3) for each individual participant, highlighting the consistency of the rightward shift in spatial bias over time across participants.

Figure 3D shows the mean curve widths from individually fitted psychometric functions (% of absolute line length) for each third of the experiment. Discrimination sensitivity for the task remained stable over the course of the experiment. A repeated‐measures anova on the curve width values with time‐on‐task as a within‐subject factor (three levels) revealed no significant main effect (*F*_2,36_ = 0.577, *P* = 0.567, $\eta_{p}^{2}$ = 0.031). To illustrate this, Fig. 3E plots the difference in curve width between the final blocks (7–9) and the initial blocks (1–3) for each individual participant.

### EEG results

While participants viewed the horizontal lines and performed the landmark task, their EEG was recorded from 60 scalp electrodes. EEG time series, epoched around the onset of each landmark stimulus, were then transformed into the spectral domain to yield time‐frequency representations of ongoing oscillatory activity. These data, in combination with a jackknife procedure, allowed us to identify those spectral features in single‐trial pre‐ (and post‐) stimulus data that co‐varied with the PSE and/or curve width estimates.

### Pre‐ (and post‐) stimulus EEG power predict spatial bias

**Figure 4:**
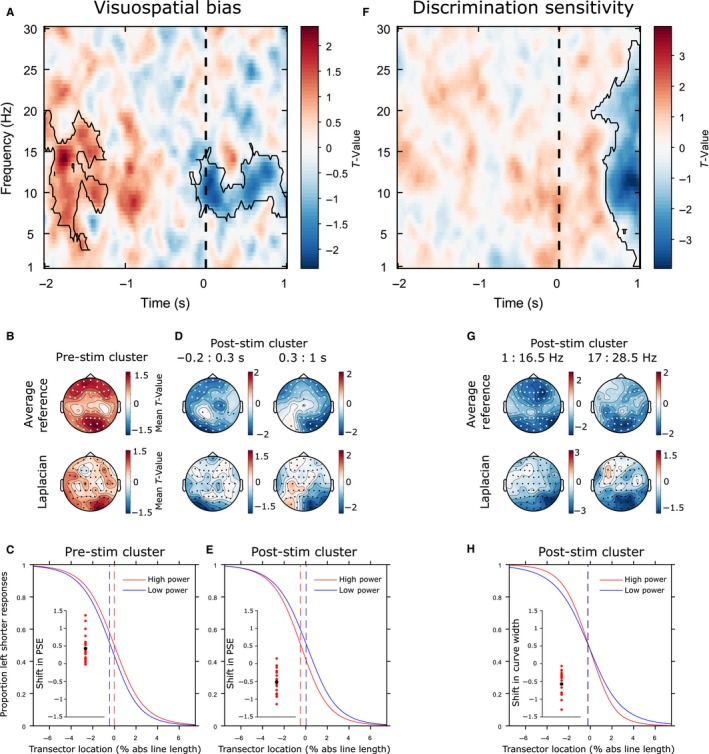
EEG results. Pre‐stimulus alpha predicts visuospatial bias (PSE) but not discrimination sensitivity (curve width). **(A)** Power‐PSE effect: T‐values averaged across all 60 electrodes. A positive *t*‐value indicates that high power is associated with relatively rightward PSEs and low power with relatively leftward PSEs. A negative *t*‐value indicates that high power is associated with relatively leftward PSEs and low power with relatively rightward PSEs. Significant clusters are outlined with a solid black line. The vertical black dashed line represents stimulus onset. (B) Topographical representations of the *t*‐values associated with the pre‐stimulus cluster (top row: electrodes that were significant at least once at any time‐frequency point within the cluster are highlighted in white). Note that cluster‐based permutation tests were not performed on the Surface Laplacian data (bottom row); rather, this topography was calculated to provide a more topographically localized estimate of the effect. (C) Group‐averaged psychometric functions (PFs) calculated separately for high and low power trials (median split averaged across all data points in the pre‐stimulus cluster; mean PSEs represented by vertical dashed lines) and differences in PSE for each individual participant (inset scatterplot). Positive values indicate a rightward shift from low to high power. Red dots represent individual participant differences. The black dot indicates the group mean difference. (D) Topographical representations of the *t*‐values associated with the post‐stimulus cluster. (E) Group‐averaged PFs calculated separately for high and low power trials (early post‐stimulus cluster) and differences in PSE for each participant. Negative values indicate a leftward shift. (F) Power‐curve width effect. A negative *t*‐value indicates that high power is associated with relatively low curve width/high discrimination sensitivity and low power with relatively high curve width/low discrimination sensitivity. Significant clusters are outlined with a solid black line. (G) Topographical representations of the *t*‐values associated with the cluster. (H) Group‐averaged PFs calculated separately for high and low power trials (late post‐stimulus cluster) and differences in curve width for each participant. Negative values indicate an increase in discrimination sensitivity from low to high power.

Figure 4A illustrates the strength of the relationship between EEG power and spatial bias (PSE) from 2s pre‐ to 1s post‐stimulus for frequencies between 1 and 30 Hz averaged across all electrodes resulting from the jackknife procedure. A positive *t*‐value (coded in red) indicates a positive relationship between EEG power and PSE value (i.e. high power associated with relatively rightward bias and low power with relatively leftward bias). A negative *t*‐value (coded in blue) indicates an inverse relationship between EEG and PSE value (i.e. high power associated with relatively leftward bias and low power with relatively rightward bias). We found two clusters (each outlined in Fig. 4A with a solid black line) that survived multiple comparison correction (5% alpha level): (1) A positive pre‐stimulus cluster (cluster statistic = 14892.48, *P* = 0.0125), and (2) a negative, mainly post‐stimulus cluster (cluster statistic = −18188.33, *P* = 0.0045).

The pre‐stimulus cluster was broadband covering the alpha and adjacent frequency bands (3–20 Hz) (Fig. 4A) and was widely distributed over the scalp with notable maxima at frontal and right posterior sites, as indicated by the topographical representation of the *t*‐values associated with the effect (see Fig. 4B). To improve topographic localization and minimize the effects of volume conduction, we also calculated the surface Laplacian (Perrin *et al*., 1989; Cohen, 2014) for each participant and then subjected this data to exactly the same jackknife analysis as for the original average referenced data. The resulting *t*‐value topographies (averaged over those time‐frequency data points identified by the average reference clusters) are shown in Fig. 4B (bottom map) revealing a right lateralized posterior maxima for the pre‐stimulus positive effect. When spectral power was relatively high at the data points of this pre‐stimulus cluster, PSE estimates were likely to be more rightward oriented than when power was low (median split: high vs. low power PSEs = −0.01 vs. −0.44 (means): *t*_18_ = 4.5952, *P* = 0.0002). Figure 4C shows group‐averaged PFs for high and low power trials (median split) and the scatterplot (inset) shows the difference in PSE between high and low power trials for each individual participant, highlighting the consistency of the rightward shift in spatial bias from low to high power. The relationship between z‐scored jackknife power and PSE measures from the peak data point of the pre‐stimulus cluster, collapsed across participants, is plotted in Supplementary Fig. S1A.

The mostly post‐stimulus cluster of negative power‐PSE relationships was in the 6.5–16 Hz frequency range and appeared to consist of two notable topographic patterns (based on visual exploration of the effect), an early topography (−0.2:0.3 s relative to stimulus onset) with more left lateralized frontal and posterior maxima (slightly left lateralized) followed by a later topography (0.3:1 s) which was right lateralized over posterior regions (see Fig. 4D). A Surface Laplacian analysis performed as above indicated a left lateralized posterior maxima for the early negative effect (Fig. 4D, bottom left map) and a right‐lateralized posterior maxima for the late negative effect (bottom right map). When spectral power was relatively high at the data points of this negative cluster, spatial bias was likely to be more leftward oriented than when power was low (high vs. low power PSEs = −0.49 vs. 0.03 (mean): *t*_18_  = −6.4191, *P* < 0.0001). Figure 4E shows group‐averaged PFs for high and low power trials (median split) and the scatterplot (inset) shows differences in PSE between high and low power trials for each individual participant, again highlighting the consistency of the leftward shift in the psychometric curve from low to high power.

The more traditional ‘high’ vs. ‘low’ power trial binning analysis revealed broadly similar results, although the significant clusters were less widespread than for the jackknife analysis (see Fig. S2A).

### No evidence that pre‐stimulus EEG power predicts discrimination sensitivity

Figure 4F illustrates the strength of the relationship between EEG power and curve width across time and frequency space averaged across all electrodes, where negative *t*‐values indicate an inverse relationship and positive values indicate a positive relationship. Cluster‐based statistics revealed one large negative cluster encompassing frequencies from 1 to 28.5 Hz in a post‐stimulus window (cluster statistic = −62801.93, *P* = 0.0005) which appeared to consist of two notable topographic patterns based on visual exploration of the effect. Topographical representations of the *t*‐values associated with the effect in low (1–16.5 Hz) and high (17–28.5 Hz) frequency ranges are shown in Fig. 4G. The surface Laplacian analysis revealed a right posterior maximum for the low frequencies (Fig. 4G, lower left) and a central posterior maximum for the high frequencies (Fig. 4G, lower right). When spectral power was relatively high at these data points, curve width estimates were likely to be small (i.e. discrimination sensitivity high) (high vs. low power width = 1.15 vs. 1.72 (mean), *t*_18_ = −7.2263, *P* < 0.0001). Figure 4H shows group‐averaged PFs for high and low power. The scatterplot (inset) shows differences in curve width between high and low power trials for each individual participant. The relationship between z‐scored jackknife power and curve width measures from the peak data point of the post‐stimulus cluster, collapsed across participants, is plotted in Fig. S1B. The median split analysis again revealed broadly similar results (see Fig. S2B).

### Mediation analysis: trial‐order contributes to the pre‐stimulus alpha power‐spatial bias relationship

To assess whether the observed pre‐stimulus relationship between EEG power and spatial bias estimates primarily follows a spontaneous pattern across trials or is rather driven by trial order, and hence may represent a neural correlate of the rightward shift in spatial bias over time (Manly *et al*., 2005; Dufour *et al*., 2007; Benwell *et al*., 2013a; Newman *et al*., 2013; Veniero *et al*., 2017, current experiment), we performed a mediation analysis.

**Figure 5:**
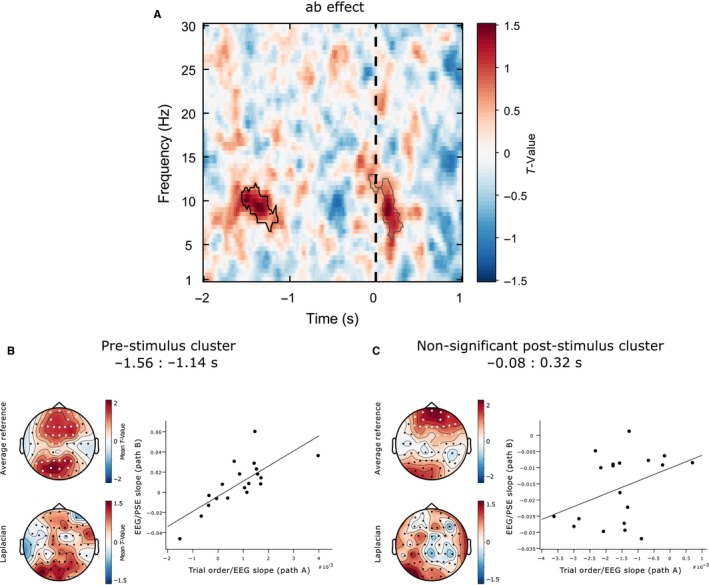
Mediation analysis. Pre‐stimulus alpha power mediates the rightward shift in spatial bias over time (time‐on‐task effect). (A) *T*‐values averaged across all electrodes at each time‐ frequency point. These *t*‐values represent a group‐level test of whether the mediation effect (*ab*) slopes from the single‐trial mediation analysis show a systematic directionality. A positive *t*‐value (coded in red) indicates that the inclusion of the mediator (oscillatory power) in the model decreased the predictive power of trial order on PSE (a classical mediation effect). Significant clusters are outlined with a solid lines. (B) Topographical representations of the *t*‐values associated with the pre‐stimulus cluster and inter‐subject correlations between the path *a* (trial order‐EEG power) and *b* (EEG power‐PSE) slope values averaged across all data points included in the pre‐stimulus cluster. (C) Topographical representations of the *t*‐values associated with the post‐stimulus cluster and inter‐subject correlations between the path *a* and *b* slope values (post‐stimulus cluster). Those participants who showed a strong relationship between trial order and pre‐stimulus alpha power (increase in alpha with time‐on‐task) were more likely to also show a strong relationship between pre‐stimulus alpha power and PSE (rightward shift from low to high power).

Figure 2 shows a diagram of our mediational hypothesis, namely that the effect of trial order on spatial bias (PSE) may be mediated by EEG oscillatory activity. Our measure of interest was the level of mediation of the trial order‐PSE relationship by EEG power. This measure (***ab***) is the product of paths ***a*** (indexing the influence of trial order on oscillatory activity) and ***b*** (indexing the influence of oscillatory activity on the PSE). Figure 5A plots t‐statistics on whether the mediation effect (***ab*** slopes) show a systematic directionality across participants, averaged over all electrodes. A positive *t*‐value (coded in red) indicates that the inclusion of the mediator ‘oscillatory power’ in the model decreased the predictive power of trial order on PSE, a classical mediation effect. We found two positive clusters (outlined in Fig. 5A with solid lines). The first, pre‐stimulus cluster (6.5–12 Hz) (cluster statistic = 2540.88, *P* = 0.0105) survived multiple comparison correction (indicated by the black solid line). A second, post‐stimulus cluster (5.5–13 Hz) (cluster statistic = 1905.46, *P* = 0.0285) just failed to survive correction (indicated by the grey solid line), but was of interest nonetheless because of its overlap with the early stage of the post‐stimulus EEG Power‐PSE relationship described above (Fig. 4A). Figure 5B,C shows topographical representations of the *t*‐values associated with the pre‐stimulus and post‐stimulus effects, respectively. The pre‐stimulus effect shows notable maxima at frontal and posterior sites, whereas the post‐stimulus effect shows frontal and left lateralized posterior maxima. The equivalent analyses performed with the surface Laplacian data revealed a posterior maximum for the pre‐stimulus effect and a left lateralized posterior maximum for the post‐stimulus effect. Alpha band power at these time points partially mediated the rightward shift in spatial bias (PSE) with time‐on‐task (trial order) (total X‐Y effect: *t*_18_ = 1.9331, *P* = 0.0691 / pre‐stimulus cluster peak (at electrode P1, 9.5 Hz, −1.5 s) X‐Y direct effect: *t*_18_ = 1.8496, *P* = 0.0809 / post‐stimulus cluster peak (at electrode FT8, 10 Hz, 0.16 s) X‐Y direct effect: *t*_18_ = 1.7482, *P* = 0.0975).

Additionally, those participants who showed a strong increase in alpha with time‐on‐task were more likely to also show a strong rightward shift in PSE from low to high power. This effect is illustrated in Fig. 5B (inter‐subject correlation between the path ***a*** and ***b*** (slope) values; Spearman's rho = 0.772, *P* = 0.00016). That is, within our two‐level mediation model in which all three variables are random effects allowed to vary across participants, the population covariance (**σ** ***ab***) of paths ***a*** (trial order‐oscillatory power) and ***b*** (oscillatory power‐PSE) was significant, further pointing to a mediation effect. Hence, for the pre‐stimulus cluster, both ***ab*** and **σ** ***ab*** were nonzero. Figure 5C shows the same inter‐subject correlation for the data points included in the post‐stimulus cluster (5.5–13 Hz) (Spearman's rho = 0.335, *P* = 0.106). Hence, for the post‐stimulus cluster, ***ab*** was nonzero but **σ** ***ab*** was not significantly different from zero.

In brief, by the overlap in space, time and frequency with the pre‐stimulus power‐PSE relationship (and to some extent also with the post‐stimulus effect), the mediation analysis indicates that co‐variation of pre‐stimulus alpha power with the PSE may represent a neural correlate of the rightward shift in spatial bias over time. This indicates the pre‐stimulus power‐PSE relationship is at least partially contingent on a deterministic variable (time‐on‐task). To ascertain whether any additional ‘stochastic’ variability in pre‐stimulus power, over and above the time‐on‐task trend, further predicts spatial bias, we performed an additional analysis. At the data point corresponding to the peak *t*‐value of the jackknife analysis pre‐stimulus cluster (14 Hz, −1.78 s at electrode AF4), we retrieved the residual variations in power after regressing out the effect of trial order within each participant. We then collapsed the data across participants and performed a stepwise regression analysis (using the ‘stepwisefit’ function in Matlab) with z‐scored (within participants) jackknife PSE estimates as the response variable and trial order and the residual variations in power as the predictor variables. The analysis revealed a significant relationship between the residual variations in power and spatial bias (β = 0.036, *t* = 3.8730, *P* = 0.0001), even after accounting for the direct influence of time‐on‐task. Hence, we find evidence that both ‘deterministic’ and ‘stochastic’ sources of pre‐stimulus EEG variability predict spatial bias, with the ‘deterministic’ aspect appearing to be restricted to a narrow, alpha‐band‐specific time‐frequency range (see Fig. 5A).

### Pre‐stimulus alpha power predicting spatial bias: No relation to lateralization index across posterior regions of interest (ROI)

**Figure 6:**
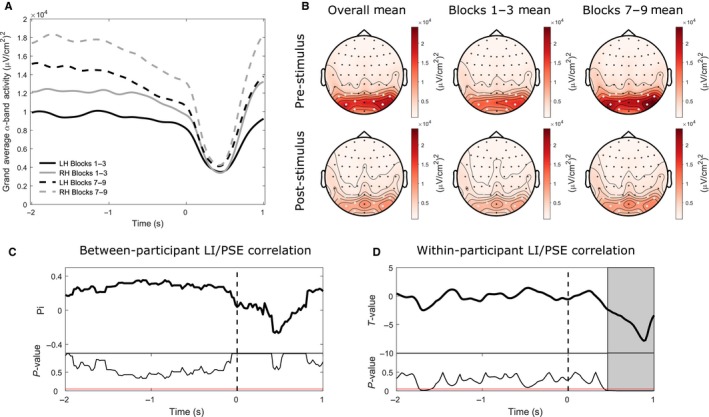
Lateralization index ROI analysis. Pre‐stimulus hemispheric lateralization of posterior alpha power does not strongly predict spatial bias either between or within subjects. (A) Group‐averaged alpha power for the right (grey lines) and left (black lines) hemisphere ROIs in both the first (solid lines) and final (dashed lines) third of the experiment. (B) Surface Laplacian topographies of alpha power averaged per pre‐stimulus and post‐stimulus time periods. The topographies in the left, middle and right columns display the overall group mean, the mean in the first blocks of the experiment (first third of all trials) and in the final blocks of the experiment (final third of all trials), respectively. Electrodes highlighted in white represent the selected left and right posterior ROIs, respectively. Alpha power was higher in the final than first blocks and was higher over the right than the left hemisphere. (C) Between‐participant correlation between mean alpha LI values and PSE across participants at all time points. The top panel displays Shepherd's pi values (robust equivalent of Spearman's Rho) and the bottom panel displays the corresponding *P*‐values. The horizontal red line represents a *P*‐value of 0.05. No significant correlations were found. (D) Within‐participant single‐trial regression between jackknife alpha LI values and jackknife PSE values. The top panel displays *t*‐values representing group‐level tests of whether regression slopes from the individual single‐trial analyses show a systematic linear relationship (i.e. are significantly different from 0) across participants and the bottom panel displays the corresponding *P*‐values. The grey background fill denotes a significant cluster‐corrected post‐stimulus correlation whereby increased right hemisphere > left hemisphere alpha power was associated with a relatively leftward bias. No significant pre‐stimulus clusters were found (in contrast to the non‐lateralized pre‐stimulus power analysis).

Given previous reports of a relationship between the relative lateralization of pre‐stimulus posterior alpha power and the horizontal distribution of spatial attention (Thut *et al*., 2006; Newman *et al*., 2013, 2016; Loughnane *et al*., 2015; Slagter *et al*., 2016), and to facilitate comparison to this literature, we performed an additional ROI analysis (see Fig. 6).

For the pre‐stimulus data, posterior alpha power was higher in the final than first blocks and was higher over the right than the left hemisphere (Fig. 6A,B). The corresponding 2 (hemisphere: left, right) x 2 (time: first third, final third) repeated‐measures anova revealed a main effect of time (*F*_1,18_ = 14.481, *P* < 0.001, $\eta_{p}^{2}$ = 0.446) and a non‐significant trend for an effect of hemisphere (*F*_1,18_ = 3.802, *P* = 0.067, $\eta_{p}^{2}$ = 0.174) but no time x hemisphere interaction (*F*_1,18_ = 0.666, *P* = 0.425, $\eta_{p}^{2}$ = 0.036). Alpha power increased from the first to the final third of the experiment (*t*_18_ = 3.805, *P* < 0.001, Cohen's *d *=* *1.171) and was higher over the right than left hemisphere, although this effect did not reach statistical significance (*t*_18_ = 1.913, *P* = 0.072, Cohen's *d *=* *0.493).

Post‐stimulus alpha power was also higher in the last than initial blocks and appeared to be higher over the RH than the LH. The corresponding 2 × 2 repeated‐measures anova revealed a main effect of time (*F*_1,18_ = 18.404, *P* < 0.001, $\eta_{p}^{2}$ = 0.506), a main effect of hemisphere (*F*_1,18_ = 8.318, *P* = 0.01, $\eta_{p}^{2}$ = 0.316) but no time x hemisphere interaction (*F*_1,18_ = 3.26, *P* = 0.088, $\eta_{p}^{2}$ = 0.153). Overall, alpha power increased from the first to the final third of the experiment (*t*_18_ = 4.29, *P* < 0.001, Cohen's *d *=* *1.894) and was higher over the right than left hemisphere (*t*_18 _= 2.812, *P* = 0.012, Cohen's *d *=* *0.754).

Looking more closely into any possible link between posterior alpha lateralization and behaviour, we performed additional correlation analyses on the lateralization index (LI) values calculated based on the posterior ROIs. Between‐participant analysis on these LI values revealed no significant correlation between mean alpha LI values and spatial bias across participants at any time point (see Fig. 6C). However, a within‐participant single‐trial regression analysis found a significant cluster‐corrected post‐stimulus correlation between alpha LI values and spatial bias within participants (see Fig. 6D) which began 0.46s after stimulus onset and continued until the end of the epoch, coinciding in time with the second negative post‐stimulus cluster revealed by the whole‐scalp jackknife analysis (cf. Fig. 4D, lower right, note the posterior left positive‐ right negative asymmetry in the corresponding Surface Laplacian map). In addition, there was a weak correlation pre‐stimulus which however did not survive cluster correction in contrast to the non‐lateralized pre‐stimulus power analysis.

Collectively, these additional analyses suggest that the pre‐stimulus relationship between alpha power and landmark task spatial bias, identified through the whole‐scalp jackknife procedure, did not seem to be driven by a relative change in lateralization of alpha power between hemispheres, while some of the post‐stimulus correlates may reflect relative hemispheric asymmetry.

### Pre‐stimulus phase vs. spatial bias and discrimination sensitivity: Null results

**Figure 7:**
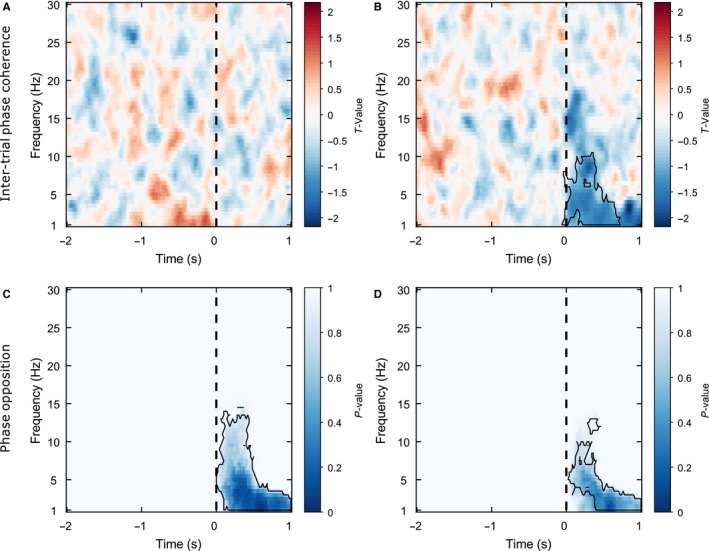
Phase Analysis. Pre‐stimulus phase does not predict either spatial bias or discrimination sensitivity. (A) Inter‐trial phase coherence (ITPC)‐spatial bias effect: *T*‐values averaged across all electrodes at each time‐ frequency point. These *t*‐values represent group‐level tests of whether regression slopes from the individual single‐trial analyses show a systematic linear relationship (i.e. are significantly different from 0) across participants. A positive *t*‐value indicates a positive relationship between ITPC and spatial bias (i.e. high phase‐locking associated with a relatively rightward subjective mid‐point and low phase‐locking with a relatively leftward subjective mid‐point). The vertical black dashed line represents stimulus onset. No significant clusters were found suggesting that ITPC does not significantly co‐vary with subjective mid‐point estimation. (B) ITPC‐curve width effect: Negative *t*‐values indicate an inverse relationship between ITPC and curve width (i.e. high inter‐trial coherence associated with relatively high discrimination sensitivity and low inter‐trial coherence with relatively low discrimination sensitivity) whereas positive *t*‐values indicate a positive relationship (i.e. high inter‐trial coherence associated with relatively low discrimination sensitivity and low inter‐trial coherence with relatively high discrimination sensitivity). A significant post‐stimulus effect was found (outlined by a solid black line), likely related to the phase alignment of the event‐related potential (ERP). (C) Spatial bias phase opposition analysis: Combined *P*‐values averaged across all electrodes at each time‐ frequency point. *P*‐values were combined from circular Watson–Williams tests performed within each participant, indicating whether ‘left’ and ‘right’ line segment shorter responses tend to be phase‐locked to different phase angles across participants. No significant pre‐stimulus effects were found suggesting that the preferred pre‐stimulus phase‐locking angle does not significantly differ between ‘left’ and ‘right’ shorter responses. Post‐stimulus preferred phase angle differed between the two responses and this is likely due to ERP differences. (D) Discrimination sensitivity phase opposition analysis. Combined *P*‐values indicate whether high and low curve width trials (estimated from a jackknife procedure) tend to be phase‐locked to different phase angles across participants. No significant pre‐stimulus effects were found suggesting that the preferred pre‐stimulus phase‐locking angle does not significantly differ between high and low discrimination sensitivity trials. Post‐stimulus preferred phase angle differed between the two outcomes and this is again likely due to ERP differences.

Figure 7A illustrates the strength of the relationship between ITPC and spatial bias across time and frequency space averaged over all electrodes, where negative *t*‐values indicate an inverse relationship and positive *t*‐values a positive relationship. No significant clusters were found (all cluster *P*‐values >0.8621) suggesting that inter‐trial phase coherence does not significantly co‐vary with subjective mid‐point estimation.

Figure 7B illustrates the strength of relationships between ITPC and curve width in time‐frequency space, where negative *t*‐values indicate an inverse relationship (i.e. high ITPC associated with relatively high discrimination sensitivity and low ITPC with relatively low discrimination sensitivity) and positive *t*‐values indicate a positive relationship (i.e. high ITPC associated with relatively low discrimination sensitivity and low ITPC with relatively high discrimination sensitivity). One significant negative cluster was found in the 1:10.5 Hz frequency band (centred at 5 Hz, i.e. theta) from −0.06:0.72 s (cluster statistic = −8643.44, *P* = 0.0095) which was distributed over the whole scalp. Hence, a decrease in curve width (increase in discrimination sensitivity) occurred from low to high post‐stimulus ITPC, but we found no evidence that the amount of pre‐stimulus ITPC predicted either subjective mid‐point estimation or discrimination sensitivity.

Moreover, an additional phase opposition analysis (results illustrated in Fig. 7C–D) found no pre‐stimulus differences in preferred phase angle between either left and right shorter responses (indexing spatial bias, Fig. 7C) or high and low discrimination sensitivity trials (Fig. 7D). However, both measures showed significant phase opposition in low‐frequency bands (PSE: ~1–15 Hz, 0–1 s, curve width: ~1:13 Hz, 0.02–2 s) post‐stimulus onset.

Hence, neither the pre‐stimulus strength nor the angle of phase coherence predicted perceptual outcome on the landmark task. In contrast, the angle of phase coherence in response to the stimulus was related to subjective mid‐point estimation. Further, both the post‐stimulus strength and the angle of phase coherence were related to discrimination sensitivity. These post‐stimulus effects were likely related to stimulus‐evoked activity (event‐related potentials).

## Discussion

We investigated the EEG time‐frequency covariates of psychometric measures, indexing spatial bias and discrimination sensitivity, respectively, derived from landmark task performance. Behaviourally, we found a systematic group‐level leftward bias (pseudoneglect) at baseline which shifted rightwards over the course of the experimental session, in line with the previously reported time‐on‐task effect (Manly *et al*., 2005; Dufour *et al*., 2007; Benwell *et al*., 2013a,2013b; Veniero *et al*., 2017). Our single‐trial EEG analysis identified both pre‐ and post‐stimulus spectral power correlates of spatial bias, primarily in the alpha band, whereas only late broadband post‐stimulus power correlated with discrimination sensitivity for the task. A mediation analysis suggested that trial‐order contributes to the pre‐stimulus alpha power‐spatial bias relationship. No evidence was found for a link between pre‐stimulus phase and either spatial bias or discrimination sensitivity. The results provide novel evidence on the pre‐stimulus predictors of visual performance measures and on the role of alpha oscillations in shaping perceptual outcome, and highlight the need to take into account deterministic (vs. stochastic) sources of trial‐by‐trial variability when interpreting links between pre‐stimulus activity and behavioural measures.

### Relationship between pre‐stimulus alpha power over the right hemisphere and spatial bias: link to models of information flow and higher order attention network interactions

Pre‐stimulus oscillatory activity in the alpha band has repeatedly been shown to be related to perceptual outcome (Ergenoglu *et al*., 2004; Thut *et al*., 2006, 2012; Van Dijk *et al*., 2008; Busch *et al*., 2009; Wyart & Tallon‐Baudry, 2009; Klimesch, 2012; Kelly & O'Connell, 2013; Capilla *et al*., 2014). Despite the bulk of evidence, there is still no consensus on the functional influence of pre‐stimulus alpha oscillations on perception. Among the different interpretations put forth, the most prominent are that alpha oscillations may act to inhibit (or ‘gate’) the flow of information into sensory cortices (Jensen & Mazaheri, 2010; Romei *et al*., 2010; Lange *et al*., 2013) or alternatively to influence the flow of information from sensory cortices to higher order areas (Palva & Palva, 2007; Van Dijk *et al*., 2008; Chaumon & Busch, 2014). Our present data help to inform these open points.

Spatial bias could either originate primarily at low levels (in line with the input gating hypothesis) or higher levels (i.e. influencing readout from lower sensory to higher order areas), with the latter scenario supporting involvement of processes beyond primary visual areas. Although the current data alone cannot inform this issue directly (due to the inherent spatial limitations of EEG), several characteristics of our data do not fit with a low‐level gating account in the context of our task. We found no evidence for the relative lateralization of pre‐stimulus posterior alpha power over the left vs. right hemisphere being predictive of spatial bias, although opposing changes in posterior alpha power over both hemispheres are often observed during left vs. rightward endogenous attention shifts (Worden *et al*., 2000; Sauseng *et al*., 2005; Kelly *et al*., 2006; Thut *et al*., 2006; Rihs *et al*., 2007; Capotosto *et al*., 2009; Gould *et al*., 2011), interpreted as a preparatory change in the excitability of low‐level visual regions (for review see Foxe & Snyder, 2011). Instead, we found the amplitude of posterior alpha power to be higher in the right than the left hemisphere overall, an effect consistently observed in other studies in the absence of attentional cueing (Slagter *et al*., 2016; Newman *et al*., 2017), indicating that our results may need to be interpreted in the light of right hemispheric functions. It therefore appears that the effect of alpha power on spatial bias observed here, and pseudoneglect as displayed on the landmark task in general, likely occurs at a processing stage beyond primary sensory areas. Note that this contrasts with a series of studies on attentional bias derived from lateralized visual detection tasks (Thut *et al*., 2006; Newman *et al*., 2013, 2017). For instance, Newman *et al*. (2017) found a correlation between the relative lateralization of pre‐stimulus alpha power over both hemispheres and visual field RT asymmetries displayed on a lateralized coherent motion detection paradigm across participants. Likewise, in an earlier study, Newman *et al*. (2013) found posterior alpha lateralization and RT asymmetries to be co‐modulated, notably by time‐on‐task. We tentatively attribute the discrepant findings of these studies (i.e. the current study vs. Newman *et al*., 2013 Newman *et al*., 2017; see also Loughnane *et al*., 2015) to have likely captured different perceptually relevant pre‐stimulus alpha sources, potentially explained by the use of different measures of spatial bias. That is, while most studies of pre‐stimulus oscillatory predictors of perception have been restricted to detection tasks with perithreshold stimuli, we here studied to what extent pre‐stimulus oscillations influence suprathreshold landmark task performance, which may have tapped into different functions than lateralized perithreshold stimulus designs.

Why should a positive relationship emerge between alpha power and spatial bias for the landmark task? We note that the surface Laplacian analysis hints at a right hemisphere (RH) locus, indicating that one explanation could be hemispheric lateralization of the effect. A common element of the above models of information flow is that enhanced alpha power indexes a decrease in neuronal excitability as the relevant areas disengage from task execution, in line with the finding that alpha power and neuronal excitability are inversely related (Romei *et al*., 2008; Haegens *et al*., 2011; Lange *et al*., 2013). For our findings, this could mean that when RH alpha power was relatively high, and hence the RH was more disengaged, pseudoneglect was reduced leading to a rightward attention shift, as compared to when RH alpha power was low. This interpretation is in line with current models of attention networks taking into account also time‐on‐task.

It has been suggested that rightward shifts in spatial attention with time‐on‐task occur due to an interaction between spatial and non‐spatial aspects of attention such as alertness/fatigue (Manly *et al*., 2005; Fimm *et al*., 2006; Dufour *et al*., 2007; Paladini *et al*., 2016, 2017; see also Newman *et al*., 2013, 2016). In Corbetta & Shulman's (2011) neuroanatomical attention model, depletion of the primarily right lateralized ‘alertness’ network (Sturm & Willmes, 2001) results in decreased recruitment of the RH dorsal frontoparietal attention network (DAN) which would be expected to reduce pseudoneglect, that is to induce a rightward shift in spatial bias. Moreover, because posterior alpha power has traditionally been associated with both attention and arousal (Cajochen *et al*., 1995; Sadaghiani *et al*., 2010; Craig *et al*., 2012; Klimesch, 2012), this activity is likely to index changes in the corresponding neuronal networks, and associated behavioural outcomes. Paladini *et al*. (2017) recently investigated DAN excitability with a twin‐coil transcranial magnetic stimulation (TMS) approach in combination with an alertness manipulation during performance of a lateralized visual exploration task. They found that states of high alertness were accompanied by higher excitability of the right compared to the left posterior parietal cortex (PPC) (a node of the DAN (Corbetta & Shulman, 2011)) whereas states of low alertness were accompanied by lower excitability of the right compared to the left PPC. Importantly, this shift from high to low alertness was accompanied by a rightward shift in bias displayed on the exploration task. Additionally, Newman *et al*. (2016) manipulated alertness levels (using blue‐enriched light) prior to performance of a lateralized detection task. They found an effect of enhanced reaction times for left (and not right) visual field targets in participants who had been pre‐exposed to high‐intensity light. This effect was mediated by a reduction of RH alpha power by high‐intensity light. Interestingly, spatial bias displayed on the landmark task has previously been linked to frontoparietal network activity and anatomy including the DAN (Szczepanksi & Kastner, 2013; Thiebaut de Schotten *et al*., 2011) and neural activity in the DAN, as measured by fMRI, has been found to negatively correlate with posterior EEG alpha power (Laufs *et al*., 2003; Sadaghiani *et al*., 2010; Chang *et al*., 2013; Zumer *et al*., 2014).

Hence, there is converging evidence for the interaction of spatial and non‐spatial aspects of attention and their corresponding neural networks in the genesis of shifts in spatial bias over time. Based on the current results, we propose that a candidate mechanism for the rightward shift in spatial bias with time‐on‐task is a downregulation of RH DAN activity over time which is indexed by an increase in posterior RH alpha power. This account is congruent with the effect of alpha on spatial bias occurring at a higher order rather than low‐level sensory processing stage. It must be acknowledged that the spatial resolution of EEG necessarily limits the interpretation of anatomical sources of the observed effects. This may be addressed by future research combining EEG with fMRI and/or TMS.

### Stochastic vs. deterministic sources of the link between pre‐stimulus oscillations and performance variability

The current study provides novel evidence that the previously observed rightward shift in spatial bias over the course of the experimental session (Manly *et al*., 2005; Dufour *et al*., 2007; Benwell *et al*., 2013a,2013b; Veniero *et al*., 2017) is partially mediated by an increase in pre‐stimulus alpha power mainly over the right hemisphere. By extension, this indicates that the link between pre‐stimulus alpha power and spatial bias is partially driven by long‐term, deterministic changes, rather than moment‐by‐moment variability. In fact, we found evidence that ‘deterministic’ sources of pre‐stimulus oscillatory variability contribute to predicting spatial bias on the landmark task in addition to stochastic fluctuations. This finding is important for the interpretation of pre‐stimulus oscillatory predictors of perception in general. Without considering potential explanatory sources of variability, interpretation of oscillatory predictors of perception to reflect stochastic trial‐by‐trial variability may only be partially warranted, or incorrect. This argument is particularly important in situations where the psychophysical measure of interest itself is not stationary over time or may be influenced by a third explanatory variable (Monto *et al*., 2008; Fründ *et al*., 2011; Doll *et al*., 2015). In line with this, Bompas *et al*. (2015) recently identified a correlation between pre‐stimulus oscillatory power (in alpha, beta and low gamma) and subsequent saccadic response time which was explained by both short‐term, stochastic (trial‐by‐trial) and long‐term, deterministic (trial order, fatigue) sources of variance with the relative degree of each contributor differing across brain regions. Additionally, recent studies provide evidence that pre‐stimulus alpha oscillations encode biases of upcoming sensory decisions induced by top‐down predictions (Mayer *et al*., 2016; Samaha *et al*., 2016) and decisions on preceding trials (De Lange *et al*., 2013). Hence, because some short and long‐term changes in EEG characteristics systematically correlate with changes in psychophysical performance, these should be taken into consideration when interpreting trial‐by‐trial oscillatory predictors of perception.

### No evidence that pre‐stimulus power predicts visual sensitivity

While we found pre‐stimulus power to positively correlate with spatial bias across trials, no pre‐stimulus correlates of discrimination sensitivity were found in any frequency band. Hence, for landmark task performance, pre‐stimulus alpha power influenced the overall perceptual bias displayed in favour of one end of the line relative to the other, but did not appear to influence the precision of the observers’ judgements (i.e. their ability to discriminate differences in the relative sizes of the two ends of the line). One simple explanation for the lack of an effect of pre‐stimulus oscillations on relative discrimination sensitivity is a lack of variance in the sensitivity measure to pick up co‐variations in EEG. For instance, for most of the trials, there may simply be too much sensory evidence (due to the suprathreshold stimuli) for small variations in baseline neuronal activity to influence decision outcome (but note that the jackknife analysis picked up EEG co‐covariates of sensitivity in the post‐stimulus window, Fig. 4F). Another conceivable explanation for the null results is that pre‐stimulus power and sensitivity may show another type of relationship than the monotonic relationship tested here (see e.g. Rajagovindan & Ding, 2011; Snyder *et al*., 2015). Although we acknowledge that null results do not provide evidence of absence, we would like to point out analogies to recent reports of differential co‐variations of pre‐stimulus alpha power with bias vs. sensitivity that align with our results. Lange *et al*. (2013) studied pre‐stimulus predictors of the double‐flash illusion (DFI) and fusion effect (FE), both based on suprathreshold stimuli. These authors found that pre‐stimulus alpha power had no influence on visual sensitivity but predicted the likelihood of one perceptual outcome (seeing two stimuli) vs. another (seeing one stimulus), regardless of whether this perception was veridical or not. Other studies that employed perithreshold stimuli broadly within a signal detection theory framework (Green & Swets, 1966) have provided converging evidence that pre‐stimulus alpha power may primarily bias perception by changing the decision criterion (Limbach & Corballis, 2016; Craddock *et al*., 2017; Iemi *et al*., 2017), and also subsequent decision confidence (Samaha *et al*., 2017), rather than changing perceptual sensitivity (Chaumon & Busch, 2014; Limbach & Corballis, 2016; Craddock *et al*., 2017; Iemi *et al*., 2017; Samaha *et al*., 2017). It appears that pre‐stimulus alpha is not primarily influencing the veracity of perception (i.e. by increasing/decreasing visual sensitivity) but rather inducing a bias towards one perceptual outcome vs. another in cases of uncertainty. Our data therefore show analogy to previous findings of a link between pre‐stimulus alpha activity and bias but not visual sensitivity.

### Post‐stimulus predictors of psychometric measures

While the emphasis of the present study is on pre‐stimulus oscillations, the results also shed light on post‐stimulus EEG covariates of psychometric measures of landmark task performance, adding to previous studies of post‐stimulus EEG signals associated with the landmark task (Foxe *et al*., 2003; Benwell *et al*., 2014a; Longo *et al*., 2015; Learmonth *et al*., 2017). However, these studies did not link EEG activity to different aspects of task performance (i.e. spatial bias and discrimination sensitivity) directly and focused on the analysis of event‐related potentials (ERPs).

The current study reveals that the degree of alpha desynchronization is predictive of the spatial bias displayed on a trial‐by‐trial basis within participants. We found spatial bias to be negatively correlated with post‐stimulus alpha power. The time periods of the effects correspond to the period of stimulus locked alpha desynchronization. When LH alpha desynchronization was strong during the early stage, spatial bias was relatively more rightward oriented than when LH alpha desynchronization was weak. The level of alpha desynchronization indexes the level of cortical responsiveness to a visual stimulus (Klimesch, 2012) and hence our results suggest that the stronger the early posterior LH stimulus‐induced activation in response to the line, the more rightward the spatial bias is likely to be. We also found evidence that this effect is partially mediating the time‐on‐task effect, though not as robustly as the pre‐stimulus alpha effect.

Additionally, the later time period of the effect (peaking at ~0.6 s post‐stimulus, corresponding to a period of alpha synchronization (see Fig. 6A)) followed a pattern of reversed alpha lateralization. High RH/low LH alpha power during this period was associated with relatively leftward spatial bias and vice versa for low RH/high LH alpha power. A region‐of‐interest analysis confirmed a significant negative correlation between posterior alpha lateralization and trial‐by‐trial spatial bias. Although the effect occurred relatively late, it may represent a lateralization of activity necessary for the decision (van Diepen *et al*., 2016), which has recently been linked to spatial bias (Newman *et al*., 2017).

The post‐stimulus correlates must be interpreted with the caveat however that physical differences in the stimuli presented on a trial‐by‐trial basis will result in differences in evoked and induced neural responses which may partially or entirely explain the observed EEG‐behaviour correlations. Nevertheless, we show that the jackknife method employed here can be used to identify both pre‐ and post‐stimulus single‐trial variability in EEG signatures that are linked to perceptual outcome. This novel approach thereby allows for detailed interrogation of the neural signatures of distinct contributors to psychophysical performance when paired with appropriate designs.

### Limitations of study design and analysis

One limitation of the current study is that participants always indicated which end of the line appeared to be ‘shortest’ with the same finger/response mapping. Hence we cannot rule out a potential influence of motor response bias on our subjective mid‐point measures. Although the topographic representations of the identified EEG/spatial bias associations do not suggest a motor origin of the effects, future studies should alternate within and/or between participants the instruction to identify either the ‘shortest’ or ‘longest’ end of the line in order to eradicate the potential influence of response bias (Toraldo *et al*., 2004).

It is also important to note that we only tested here for linear relationships between our EEG and psychophysical measures. The literature on alpha power predictors of performance in the visual domain has consistently shown evidence for a linear relationship between EEG and behaviour mostly with data binning methods (Thut *et al*., 2006; Chaumon & Busch, 2014; Limbach & Corballis, 2016; Iemi *et al*., 2017) suggesting that this is a reasonable starting point. However, previous studies have also found non‐monotonic relationships between pre‐stimulus oscillatory power and post‐stimulus‐evoked neural activity and/or perception, primarily for tactile perception tasks (Linkenkaer‐Hansen *et al*., 2004; Zhang & Ding, 2010; Lange *et al*., 2012; Ai & Ro, 2014) but also with some evidence in the visual domain (Rajagovindan & Ding, 2011; Snyder *et al*., 2015). For instance, there is converging evidence that an intermediate level of pre‐stimulus alpha power is optimal for detection of threshold tactile stimuli, with performance dropping off for trials with lowest and highest alpha power. The results of the current study do not rule out the possibility that such a relationship may also exist between oscillatory power and our psychophysical measures of interest.

## Competing interests

The authors declare no competing interests.

## Author contributions

C.S.Y.B designed research, performed research, analysed data and wrote the article. C.K and J.G analysed data and wrote the article. M.H designed research and wrote the article. G.T designed research, analysed data and wrote the article.

## Data accessibility

All data are available upon request.

## Supporting information

Fig. S1 plots the relationships between jackknife single‐trial estimates of EEG power and both spatial bias (PSE: 1A) and discrimination sensitivity measures (curve width: 1B) from the data points corresponding to the peak *t*‐values of the respective cluster‐analysis effects (PSE peak data point: 14 Hz, −1.78 s at electrode AF4; Curve width peak data point: 9.5 Hz, 0.98 s at electrode P8).
Fig. S2 A plots the resulting *t*‐values averaged across all electrodes from the median split PSE analysis.
