# Supplementary material for: Trial‐by‐trial co‐variation of pre‐stimulus EEG alpha power and visuospatial bias reflects a mixture of stochastic and deterministic effects
Source: Eur J Neurosci. 2017 Sep 28;48(7):2566–84. doi: 10.1111/ejn.13688 (PMC6221168; doi:10.1111/ejn.13688)
Supplement: Supplementary file 2 — Fig. S1 plots the relationships between jackknife single‐trial estimates of EEG power and both spatial bias (PSE: 1A) and discrimination sensitivity measures (curve width: 1B) from the data points corresponding to the peak t‐values of the respective cluster‐analysis effects (PSE peak data point: 14 Hz, −1.78 s at electrode AF4; Curve width peak data point: 9.5 Hz, 0.98 s at electrode P8). Fig. S2 A plots the resulting t‐values averaged across all electrodes from the median split PSE analysis. [file EJN-48-2566-s002.docx]

**Supplementary material for**

**Trial-by-trial co-variation of pre-stimulus EEG alpha power and visuospatial bias reflects a mixture of stochastic and deterministic effects**

**Christopher S.Y. Benwell^1*^, Christian Keitel^1^, Monika Harvey^2^, Joachim Gross^1^, Gregor Thut^1^**

^1^ Centre for Cognitive Neuroimaging, Institute of Neuroscience and Psychology, University of Glasgow, Glasgow, UK

^2^ School of Psychology, University of Glasgow, Glasgow, UK

^*^Corresponding author: [Christopher.Benwell@Glasgow.ac.uk](mailto:Christopher.Benwell@Glasgow.ac.uk)

**Relationship between EEG power and psychometric measures**


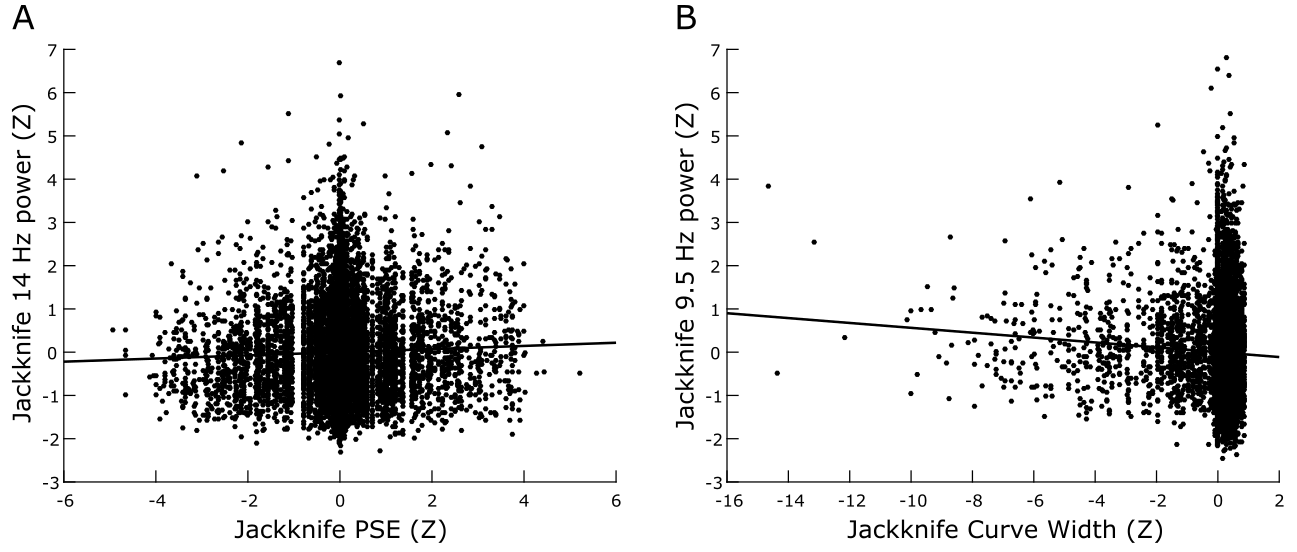


Supplementary Figure 1 plots the relationships between jackknife single-trial estimates of EEG power and both spatial bias (PSE: 1A) and discrimination sensitivity measures (curve width: 1B) from the data points corresponding to the peak t-values of the respective cluster-analysis effects (PSE peak data point: 14 Hz, -1.78 secs at electrode AF4; Curve width peak data point: 9.5 Hz, 0.98 secs at electrode P8). The single-trial data points are collapsed across all subjects with the z-score of each data point being calculated within subjects to facilitate group-level plotting. Solid black lines represent the least-squares linear fit. Linear regression analyses confirmed significant relationships between single-trial 14 Hz EEG power and PSE estimates (β = .036, F(1,11362) = 15.153, p = .0001) and between single-trial 9.5 Hz EEG power and curve width estimates (β = -.056, F(1,11362) = 34.2524, p < .0001).

We noted that the distribution of jackknife curve width values is skewed and so we performed an additional control analysis to compare the single trial jackknife approach with a more traditional binning (median split) approach which avoids the use of single-trial regression. At each data point (all times, frequencies, electrodes), we separated single-trials into ‘low’ and ‘high’ power bins for each participant (i.e. creating median split bins). We then fitted psychometric functions to the landmark task responses and retrieved PSE and curve width values separately for all trials in the ‘low’ and ‘high’ bins respectively at each data point. At the group-level, paired-sample t-tests were performed to test for systematic differences in PSE and curve width between low and high power conditions across participants at all data points, and the same cluster-based permutation testing procedure was employed as in the main jackknife analysis. As well as providing a sanity check for the results from the single-trial jackknife analysis, the median split analysis allowed us to compare the sensitivity of the two approaches.


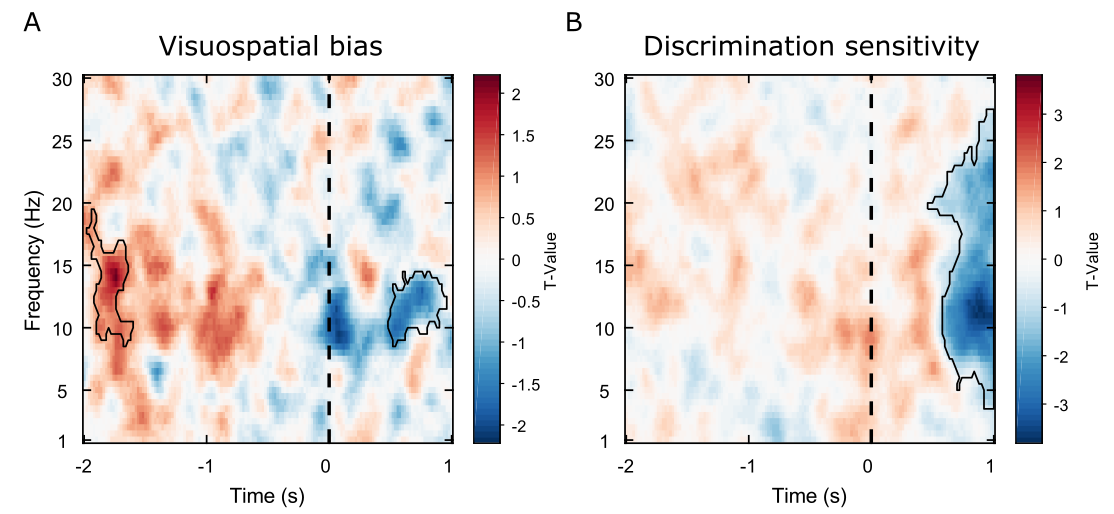


Supplementary Figure 2A plots the resulting t-values averaged across all electrodes from the median split PSE analysis. In line with the jackknife analysis, two clusters were found (each outlined in Supplementary Figure 2A with a solid black line) which survived multiple comparison correction (5% alpha level): (1) A positive pre-stimulus (cluster statistic = 4144, p = 0.0245), and (2) a negative post-stimulus cluster (cluster statistic = -5153.2, p = 0.017). The clusters were largely overlapping (in terms of times, frequencies and electrodes (topographies not shown)) with those detected by the jackknife analysis, though notably smaller (compare to Figure 4A in main manuscript). This suggests that the jackknife single-trial analysis may provide increased sensitivity compared to a median split binning approach.

Supplementary Figure 2B plots the resulting t-values averaged across all electrodes from the median split curve width analysis. Again, this analysis confirmed the results from the jackknife analysis, revealing no pre-stimulus predictor of discrimination sensitivity and one large post-stimulus, negative cluster (cluster statistic = -50186, p = 0.0005). Hence, the results from the jackknife analysis were in agreement with the median split approach despite the data being potentially sub-optimal for single-trial linear regression due to the skew of the jackknife curve width estimates.
