# Supplementary material for: Trial‐by‐trial co‐variation of pre‐stimulus EEG alpha power and visuospatial bias reflects a mixture of stochastic and deterministic effects
Source: Eur J Neurosci. 2017 Sep 28;48(7):2566–84. doi: 10.1111/ejn.13688 (PMC6221168; doi:10.1111/ejn.13688)
Supplement: Supplementary file 1 [file EJN-48-2566-s001.pdf]

EJN European Journal of Neuroscience FENS Federation of European Neuroscience Societies

Trial-by-trial co-variation of pre-stimulus EEG alpha power and visuospatial bias reflects a mixture of stochastic and deterministic effects

Christopher S.Y. Benwell, Christian Keitel, Monika Harvey, Joachim Gross & Gregor Thut

**Review timeline:**	Submission date:	18 April 2017
	Editorial Decision:	14 May 2017
	Revision received:	31 July 2017
	Editorial Decision:	23 August 2017
	Revision received:	25 August 2017
	Accepted:	25 August 2017

Editor: John Foxe

1st Editorial Decision 14 May 2017

Dear Dr. Benwell,

Your manuscript has been evaluated by three expert reviewers and the editors. As you will read below, the reviewers see significant merit in the work but also raise a number of concerns and criticisms that will require your attention in a revision before we can proceed with your paper. Questions are raised about the interpretation of null effects and whether this is justified in the absence of power analyses. Certainly, any strong claims should be tempered accordingly. Please also be careful not to interpret non-significant effects. There are calls for additional analyses that may be revealing and could add to an already very solid paper. For example, why is there no analysis of response speeds? There are also suggestions for further interpretations of your findings that will deserve your attention. Please add your treatment of phase to the main manuscript.

If you are able to respond fully to the points raised, we would be pleased to receive a revision of your paper within 8 weeks.

Thank you for submitting your work to EJN.

Kind regards,

John Foxe & Paul Bolam
co-Editors in Chief, EJN

Reviews:

Reviewer: 1 (Ulrich Pomper, University College London, UK)

Comments to the Author
The study by Benwell et al. investigates the impact of pre-stimulus neural oscillations on the subsequent perception and processing of visual stimuli.
While this question has been frequently addressed before, the authors take a novel approach by using supra-threshold visual stimuli on which a perceptual task has to be performed (the landmark task). Further, they employ a recently proposed Jackknife analysis procedure to correlate single-trial behavioural data with intertrial-phase coherence measurement, usually obtained at an across-trial level. Finally, they use mediation analysis to show that part of the previously reported correlation between pre-stimulus activity and perceptual outcomes might be related to deterministic, long term rather than trial-by trial variations in neural activity.
This study investigates a very interesting and important aspect of the relationship between neural activity and perception. The results are important, well presented, and certainly interesting to a broad readership. The paradigm is well designed and suited for the question of interest, and the performed analyses are appropriate.

I still have a few questions, comments and suggestions for improvement, and suggest the authors revise their manuscript accordingly.

Major points:
1. Given the prominence of the previously reported effects of pre-stimulus oscillatory phase on subsequent perception, I would highly suggest the authors move the results and discussion of the phase analysis, including the respective figure, into the main manuscript.

2. To me the difference between ab and σab was note made entirely clear. The manuscript would be become more accessible if the authors could try to rephrase or expand on this point.

3. The main effect of hemisphere in the pre-stimulus alpha power ANOVA is, according to the reported numbers, not significant ($p = .067$).

Minor points:
1. In the methods section, the presentation of the fixation cross is reported to be 3 s prior to the stimulus, plus 0.5 s after the response. Figure 1 displays a period of 3.5 s (including both periods). The trial design would be easier to understand if the way the intervals are reported would be consistent.

2. The legend to Figure 1 contains a lot of information which is not necessary for understanding the setup itself, all of which is included very similarly in the Methods section. I would shorten the legend to facilitate a quick understanding of the Figure.

3. p15: Figure 5A should be Figure 4A.

4. Figure 5 (presumably 4) is mentioned prior to Figures 2 and 3.

5. The difference between c and c' is not clear from Figure 4A.

6. Since the use of a surface Laplacian is not as common in the EEG literature as the other methods employed (e.g. time-frequency transformation), the authors should add a few references exemplifying the use of this method in similar situations.

7. Similarly, the authors should add references and a motivation for the specific electrodes used in the occipital ROI for the alpha-analysis.

8. The authors should elaborate on how (based on what criteria) they separated the significant time-frequency cluster presented in Figure 3D and 3G into the respective 2 sub-clusters.

9. The abbreviations PF, LH and RH are not defined in the text.

10. The word 'non-significant' should be added to Figure 4 when mentioning the second (post-stimulus) cluster.

11. The authors use the terms ITC, ITPC and phase-locking on various occasions in the manuscript. They should settle on one term to make the manuscript easier to read.

Reviewer: 2 (Adam Snyder, University of Pittsburgh, USA)

Comments to the Author
The power and phase of pre-stimulus alpha-band oscillations has been linked to perceptual performance. However, improvements in task hit rates could be accounted for by shifts in either sensitivity or criterion (i.e., response bias). The authors tasked subjects with a line bisection task while recording scalp EEG to ask which of these two potential changes in performance could be linked to shifts in pre-stimulus alpha-band power. They conclude that shifts in alpha band power are related to shifts in response bias, but they did not find evidence that shifts in alpha band power are related to changes in sensitivity. This does seem like an important and timely question. For example, a recent report from Luo and Maunsell (2015, Neuron) found that neural correlates of attention in extrastriate visual cortex could only be attributed to sensitivity changes, rather than shifts in criterion. Although the current authors rightly do not make strong claims regarding source localization, their report would seem to be a counterexample to the claims of Luo and Maunsell (admitting substantial differences between the studies), which is interesting. I do have some concerns about the current manuscript, however. Primarily I take issue with the authors' embrace of the null hypothesis (especially in the absence of a power analysis) and I wonder whether the authors might have

missed a potential non-monotonic relationship between alpha-band oscillations and task performance.

Primary concerns:

1) The title states that pre-stimulus alpha power "...does not influence discrimination sensitivity." The authors are basing this claim on the fact that they failed to find a relationship between alpha power and sensitivity. However, failure to find an effect is not the same as positive evidence for the absence of an effect. The authors should temper their language accordingly. Moreover, the inclusion of a power analysis could bolster the claim. For example, what would be the weakest relationship between alpha power and sensitivity that the authors would be powered to detect at the 80% level?

2) The authors' analysis tests for a monotonic relationship between alpha-band power and task performance. However, several researchers have found non-monotonic relationships between alpha-band power and task performance (e.g., Ai & Ro, 2014, J Neurophysiol; Linkenkaer-Hansen et al., 2004, J Neurosci; Zhang & Ding, 2010, J Cogn Neurosci; Snyder et al., 2015, Nat Neurosci; see also, Rajagovindan & Ding, 2011, J Cogn Neurosci). The authors should test for this possibility.

Lesser concerns:

3) With respect to the potential influence of the phase of neural oscillations on perceptual performance, the authors might consider referring to the work of Ian Fiebelkorn (e.g., Fiebelkorn et al., 2011, J Neurosci; Fiebelkorn et al., 2013).

4) There are two “Benwell et al., 2014” papers in the reference list, but there is no ‘a’ nor ‘b’ to enable me to tell which paper is meant when the authors cite “Benwell et al., (2014b)” on page 6.

5) Since the authors find a new set of regression coefficients at each time-frequency-channel data point, the formula on page 13 may be more accurate if the beta terms are also indexed over time and frequency.

6) What do the authors mean by “condition labels” on page 14? Does this refer to which stimulus was shown, what the subjects’ responses were, both, or something else? Why was the shuffling done only for a subset of the subjects on each iteration? How large was the subset? I would think leaving some of the subjects' data unshuffled would be overly conservative...

7) On page 15 the authors reference Figure 5, but I think from the context that they mean Figure 4 here.

8) What, exactly, was done for time-frequency transformation? The authors say on page 10 that they used a 0.5 s window and achieved 0.5 Hz frequency resolution. However if this was just short-time Fourier transformation then the frequency resolution should be 2 Hz for a 0.5 s window. Was this using the multitaper method? Something else (ft_freqanalysis is basically just a wrapper that supports several methods)?

9) On page 9 it says 62 scalp electrodes were used, but on page 19 it says that 60 scalp electrodes were used. Please clarify.

10) Please expand the acronym “PFs” on first use (p. 18).

11) When the authors state that effects “appeared to consist of two notable topographic patterns” (p.20, 21), how was this determination made? Just by eyeing the data, or was a more principled approach used somehow (e.g., microstate analysis)?

12) The Baron and Kenny procedure specifies that there should first be a significant relationship between X and Y to be mediated, but the total effect by the authors is reported as “$t(10) = 1.9331$, $p = 0.0691$” (p. 23), which is marginal. How valid is this mediation analysis if there is not clearly an effect to mediate?

13) The authors examined whether lateralization of alpha power was related to the response bias, but what about the same analysis for sensitivity?

14) The legend to Figure 1 says that the next trial started as soon as the response was made, whereas the Methods section says that the next trial started 0.5 s later. Please clarify.

15) I would like to have a better sense of the measures of interest. Would it be possible to include a distribution over jackknife repetitions of the bias and sensitivity estimates (and ITPC in the supplement)? I am wondering whether there is sufficient variation in sensitivity that an effect could be found. In other

words, could one reason why the authors did not find pre-stimulus alpha-band power was related to sensitivity be because sensitivity didn't vary much during this task?

16) More generally, the first data figure is already quite processed, showing only t-statistics for the distributions of regression coefficients: I would like to see some data in more "raw" forms, illustrating the intermediate analysis steps (e.g., a scatter plot comparing PSEs to alpha power, or something similar).

17) The axes labels for the inset panels in Figure 3 are too small to be legible.

18) The legend for Figure 3 states that significant electrodes are shown in white. Does that imply that every single electrode in the top of panel B is significant (including those that have a mean T-score of 0), and none of the electrodes in the bottom of panel B is significant? Everything (or very nearly) is white in the top row of the topographies, and everything is black in the bottom row. Is this right?

19) When the authors compute surface Laplacians, this is done on the data before time-frequency transformation, correct? It could be possible, albeit improper, to do the analysis in the other order…

20) In Figure 4, alpha band activity is given in units of uV/cm^2. However, the Methods section states that power values were expressed in decibels. Are the units right here? Even without the decibel transformation, the formula on page 10 would give squared microvolts (per square centimeter, for the Laplacian).

Reviewer: 3 (Simon Kelly, University College Dublin, Ireland)

Comments to the Author
In this study, Benwell et al examine the relationship between pre-stimulus alpha-band power and visuospatial bias and sensitivity in a landmark task. They support previous work in showing a relationship with bias but not sensitivity, and go further to show in a mediation analysis that this relationship could at least partially be driven by time-on-task effects. Both overall alpha and alpha lateralization were tested, and interestingly only the former correlated with perceptual bias.
The paper is written very well, with detailed and thorough methods and a comprehensive display of the results. My comments below relate to a couple of additional analyses that would be easy to do, but which the authors may have good reasons not to do and could clarify, and with going a little further in interpretation.

The subjects responded with their index versus middle finger to indicate their perception of the left versus right part of the line being shorter. Can the authors rule out that it could be a motor bias rather than a perceptual bias?
Related to this, I was curious as to why the authors did not examine RT, since they mention a good deal of previous work showing effects of prestimulus alpha on RT. RT is presumably also something that could change with time on task, and may well be asymmetric for the two fingers used, which have a fixed mapping to the two sensory alternatives.

Some of the interpretations are not fully clear in the paper as it currently stands. For example, I'm not sure that the mediation analysis has been interpreted fully and clearly – I see that it indicates that not all of the influence of time-on-task on PSE is direct, and that some of this is mediated through alpha, but what does it mean regarding the relationship between alpha and spatial bias itself? The paper is motivated by the need to distinguish "deterministic" versus "stochastic" sources of variability in alpha which may predict bias, but I'm still not sure after reading the paper how much of these two kinds of variability are in play in the data. It seems that in order to know whether it's not all time-on-task, i.e., that there is some additional variability in baseline alpha over and above the time on task trend, which further predicts bias, the authors would have to do a stepwise regression, i.e. once the direct influence of time on task is accounted for, does adding the additional independent variable of alpha (perhaps most appropriately the residual variations in alpha after separately regressing out the effect of time on task) explain yet more variance in the behavioural bias?
Another matter that could use more discussion is the nature of this alpha relationship with bias. Specifically, what determines the direction of the bias? Why might greater alpha specifically relate to rightward as opposed to leftward bias in the current task? The authors suggest that an asymmetry in baseline excitability in neurons coding for one alternative versus the other might underlie the alpha effects, but this seems at odds with the fact that there was no spatially specific component of alpha that showed this relationship. That is, there is no evidence for alpha reflecting any kind of asymmetry in itself (end of page 28).
Finally, the bias-predictive alpha activity comes and goes quite a long time before the stimulus onsets. Can the authors discuss why this might be? Most pre-stimulus effects tend to last right up until the stimulus actually occurs – is there some explanation for why this might not be the case here?

A general point on writing style: There is an overuse of parantheses throughout the paper. For clarity, I suggest writing additional full sentences rather than trying to cram too many points into each individual sentence. Also, the authors litter the paper with "see above" and "see below" statements, and so it's quite

jarring to follow the narrative. I suggest choosing a sequence of ideas that can proceed linearly so that it isn't necessary to refer back and refer forward quite so much. Sometimes this can't be helped, but in cases like the second paragraph of the discussion, this is both inconvenient and maybe even misleading for the reader. "Our data suggest that posterior pre-stimulus alpha fluctuations can shape perception through influencing decision bias at higher order processing stages, potentially during readout of sensory evidence from sensory cortex, as discussed below" is actually quite a major claim and really needs to be justified immediately, not put off to several sections later. The claim itself could be postponed until the narrative is ready to address it fully.

Minor:
In the intro (p3), Kelly et al (2006) is cited in relation to alpha lateralization predicting RT, but I think the authors may mean Kelly et al (2009) in this instance.

Top of page 4 (intro): could the authors explain what they mean by "higher-order" in describing the task? I'm not sure what this might mean.

The paragraph starting "We here expand" is somewhat awkwardly worded and could use editing for clarification. The previous paragraph ends with the statement that bias, and not sensitivity, has been linked to prestimulus alpha; then this one starts talking about "two separate psychometric measures," as if two new concepts are about to be introduced, but which turn out to be bias and sensitivity. I suggest first defining bias and sensitivity and how they are "separate," then to cut straight to the new element of this study, which is to measure them in the landmark task.

Methods:
Task: "The subsequent trial began 0.5 s after the response was made" - this seems to contradict the figure 1 legend. The numbers do add up, but the authors should state the timings consistently to avoid confusion.

Do JKRi and si (small case 's') refer to the same thing? If so, use just one of them.

Authors' Response 31 July 2017

We wish to thank the reviewers for their positive appraisals of our study and for their thorough and insightful reviews. In light of the constructive comments, we have now substantially rewritten large parts of the manuscript and have changed the title. We believe that the manuscript has now been considerably improved by the input of all three reviewers. Below, we have responded to each point. Changes to the text of the manuscript are underlined throughout.

Reviewer: 1

Comments to the Author

The study by Benwell et al. investigates the impact of pre-stimulus neural oscillations on the subsequent perception and processing of visual stimuli.

While this question has been frequently addressed before, the authors take a novel approach by using supra-threshold visual stimuli on which a perceptual task has to be performed (the landmark task). Further, they employ a recently proposed Jackknife analysis procedure to correlate single-trial behavioural data with intertrial-phase coherence measurement, usually obtained at an across-trial level. Finally, they use mediation analysis to show that part of the previously reported correlation between pre-stimulus activity and perceptual outcomes might be related to deterministic, long term rather than trial-by trial variations in neural activity.

This study investigates a very interesting and important aspect of the relationship between neural activity and perception. The results are important, well presented, and certainly interesting to a broad readership. The paradigm is well designed and suited for the question of interest, and the performed analyses are appropriate.

I still have a few questions, comments and suggestions for improvement, and suggest the authors revise their manuscript accordingly.

Major points:

1. Given the prominence of the previously reported effects of pre-stimulus oscillatory phase on subsequent perception, I would highly suggest the authors move the results and discussion of the phase analysis, including the respective figure, into the main manuscript.

Thank you, we have now moved the phase analysis into the main manuscript.

2. To me the difference between ab and σab was note made entirely clear. The manuscript would be become more accessible if the authors could try to rephrase or expand on this point.

We apologise for not making this clear enough and have attempted to clarify the point with the following addition beginning on page 16 (including a relevant citation to Kenny et al., 2003):

*"To clarify the difference between **ab** and **σab**, **ab** represents the degree to which the X-Y relationship is reduced **within** a given participant when the proposed mediator is controlled for, whereas **σab** is the correlation between paths a and b **across** participants (Kenny et al., 2003). In our case, **ab** represents the reduction in the trial order-PSE relationship when changes in oscillatory power over trials are controlled for and **σab** represents the correlation between the strength of the trial order-alpha power relationship and the strength of the alpha power-PSE relationship across participants. Both of these measures can signal the existence of mediation because there are two ways in which the direct effect (**c'**) might be less than the total effect (**c**) in a multilevel mediation model. First, there is reduction if **ab** is systematically nonzero across participants, or second, if the between subject correlation between paths a and b (**σab**) is nonzero (or a combination of both)."*

We hope that the difference between ab and σab has been made fully clear by this insertion.

3. The main effect of hemisphere in the pre-stimulus alpha power ANOVA is, according to the reported numbers, not significant ($p = .067$).

Thank you, we have now explicitly stated that the effect did not reach significance in the results section.

Minor points:

1. In the methods section, the presentation of the fixation cross is reported to be 3 s prior to the stimulus, plus 0.5 s after the response. Figure 1 displays a period of 3.5 s (including both periods). The trial design would be easier to understand if the way the intervals are reported would be consistent.

We apologise for this oversight. Figure 1 has now been amended accordingly in order to exactly display the trial procedure.

2. The legend to Figure 1 contains a lot of information which is not necessary for understanding the setup itself, all of which is included very similarly in the Methods section. I would shorten the legend to facilitate a quick understanding of the Figure.

We have amended the Figure 1 legend by deleting extraneous information that is already reported in the method section. We now believe it is easier to understand the figure.

3. p15: Figure 5A should be Figure 4A.

Thank you for noticing this, the text has now been amended accordingly. The figure numbers have now changed based on comment 4 below and all figures are now correctly referred to in the text.

4. Figure 5 (presumably 4) is mentioned prior to Figures 2 and 3.

The reviewer was correct to point this out. We have now created a stand-alone figure for the mediation model and this is now figure 2. Hence all of the subsequent figures have been relabelled (i.e. the old Figure 2 is now Figure 3 etc.).

5. The difference between c and c' is not clear from Figure 4A.

We have now specified (in brackets) in the new Figure 2 what c and c' represent (total effect and direct effect respectively) as well as specifying that ab represents the indirect effect. Thank you for this suggestion.

6. Since the use of a surface Laplacian is not as common in the EEG literature as the other methods employed (e.g. time-frequency transformation), the authors should add a few references exemplifying the use of this method in similar situations.

Thank you for this suggestion, we have now moved the citations associated with use of the surface Laplacian up to the first time the concept is introduced (page 17) and have also added a more detailed description of the surface Laplacian along with further citations of papers which have successfully employed the surface Laplacian to isolate distinct attention related left and right hemisphere alpha clusters:

"In order to try to improve topographic localization of alpha power separately for the left and right hemispheres, this analysis was performed on data calculated with a surface Laplacian (Perrin et al., 1989, Cohen, 2014). The surface Laplacian, a term which is interchangeable with current source density (CSD), is a spatial filter which yields topographies with reduced volume conductance (Kayser and Tenke, 2015). This allows for improved topographic localisation of underlying cortical sources of rhythmic brain activity (Keitel et al., 2013, 2017, see also Tenke and Kayser, 2015)."

7. Similarly, the authors should add references and a motivation for the specific electrodes used in the occipital ROI for the alpha-analysis.

The relevant citations have been added on page 17.

8. The authors should elaborate on how (based on what criteria) they separated the significant time-frequency cluster presented in Figure 3D and 3G into the respective 2 sub-clusters.

The decision to separate the clusters for visualisation purposes was based on visual exploration of the topographic representations of the clusters, and the local minima in significance between the early and late negative "blobs" of this cluster. We now specify this in the text with the following:

"The mostly post-stimulus cluster of negative power-PSE relationships was in the 6.5-16Hz frequency range and appeared to consist of two notable topographic patterns (based on visual exploration of the effect)".

Please note that we thought it useful to show the 2 topographies based on the local significance minima but we acknowledge that any conclusion derived from this observation is more indicative

than conclusive. Please also note that these two topographies are not a crucial part of our conclusions.

9. The abbreviations PF, LH and RH are not defined in the text.

We apologise for this oversight and have now defined these abbreviation on page 19 (PFs) and page 17 (LH and RH), and again in the relevant discussion section where we employ this abbreviation recurrently.

10. The word ‘non-significant’ should be added to Figure 4 when mentioning the second (post-stimulus) cluster.

Thank you, this has now been added. Note that the original Figure 4 has been renumbered 5.

11. The authors use the terms ITC, ITPC and phase-locking on various occasions in the manuscript. They should settle on one term to make the manuscript easier to read.

Thank you for this suggestion, we agree that this inconsistency was somewhat confusing and have now settled on the use of inter-trial phase coherence (abbreviated often to ITPC) throughout the manuscript.

Reviewer: 2

Comments to the Author

The power and phase of pre-stimulus alpha-band oscillations has been linked to perceptual performance. However, improvements in task hit rates could be accounted for by shifts in either sensitivity or criterion (i.e., response bias). The authors tasked subjects with a line bisection task while recording scalp EEG to ask which of these two potential changes in performance could be linked to shifts in pre-stimulus alpha-band power. They conclude that shifts in alpha band power are related to shifts in response bias, but they did not find evidence that shifts in alpha band power are related to changes in sensitivity. This does seem like an important and timely question. For example, a recent report from Luo and Maunsell (2015, Neuron) found that neural correlates of attention in extrastriate visual cortex could only be attributed to sensitivity changes, rather than shifts in criterion. Although the current authors rightly do not make strong claims regarding source localization, their report would seem to be a counterexample to the claims of Luo and Maunsell (admitting substantial differences between the studies), which is interesting. I do have some concerns about the current manuscript, however. Primarily I take issue with the authors' embrace of the null hypothesis (especially in the absence of a power analysis) and I wonder whether the authors might have missed a potential non-monotonic relationship between alpha-band oscillations and task performance.

Primary concerns:

1) The title states that pre-stimulus alpha power "...does not influence discrimination sensitivity." The authors are basing this claim on the fact that they failed to find a relationship between alpha power and sensitivity. However, failure to find an effect is not the same as positive evidence for the absence of an effect. The authors should temper their language accordingly. Moreover, the inclusion of a power analysis could bolster the claim. For example, what would be the weakest relationship between alpha power and sensitivity that the authors would be powered to detect at the 80% level?

We fully agree with the reviewer that failure to find an effect is not evidence for the absence of an effect and so we have now rewritten parts of the manuscript in order to tone down interpretation of the null finding with regard to discrimination sensitivity. Accordingly, we have also changed the title of the paper in order to eliminate the emphasis on this null result.

As part of rewriting to temper our language, we have moved the discussion on the null result in terms of pre-stimulus power versus sensitivity towards the end of the discussion section. Please see the new paragraph entitled: "Null result: No evidence for pre-stimulus power predicting visual sensitivity", where we emphasize conceivable alternative explanations of the null result (e.g. reduced variation in the sensitivity measure, your point 15 below) to provide a more balanced (appropriate) discussion of this null result. As a consequence, we do not embrace any conclusion based on the null result (only pointing out the analogy between our and previous findings in terms of pre-stimulus alpha signatures of bias vs sensitivity which we think is interesting and worthwhile to present).

As part of the associated overhaul of the discussion section, we also have moved all sections on alpha and spatial bias (our positive finding) into the first discussion paragraph, now entitled "Relation of pre-stimulus alpha-power over the right hemisphere to spatial bias: Link to models of information flow and higher-order attention networks".

2) The authors' analysis tests for a monotonic relationship between alpha-band power and task performance. However, several researchers have found non-monotonic relationships between alpha-band power and task performance (e.g., Ai & Ro, 2014, J Neurophysiol; Linkenkaer-Hansen et al., 2004, J Neurosci; Zhang & Ding, 2010, J Cogn Neurosci; Snyder et al., 2015, Nat Neurosci; see also, Rajagovindan & Ding, 2011, J Cogn Neurosci). The authors should test for this possibility.

Thank you for noting this relevant issue. We agree that this is a caveat to the interpretation of the current findings. We therefore have added a paragraph to the discussion stating that our analysis does not exclude the possibility that non-monotonic relationships may exist between our EEG and psychophysical measures, and briefly point to future opportunities of the Jack-knife procedure to test for them. This is now added on page 37 (see below for text). In addition, we now carefully avoid any explicit claim about the nature of the relationship (e.g. linear, monotonic etc) throughout the results section and discussion.

Please note that we have toned down the interpretation of the null-result in terms of finding no predictors of sensitivity (your point 1) also in light of this important comment, i.e. that it is conceivable that other than monotonic relationships may determine the power-sensitivity relationship.

However, we feel that investigating this issue with additional analyses would considerably overload the paper which is already of considerable length as it already includes multiple analyses to make our points on alpha-power (e.g. mediation analysis, control analysis for lateralization effects and now also analysis of phase as per reviewer 1's suggestion). Adding analyses to test for non-monotonic relationships would require substantial re-analysis within a different analysis framework and the addition of lengthy new analyses to those already presented. We hope the reviewer endorses our revisions (emphasis and interpretation of positive power-bias results, overhauling the discussion section not to embrace the null result, detailed discussion of limitations) to address his/her important points.

In addition to reorganizing the discussion section, we have added the following text to the manuscript in a new section of the discussion entitled "<u>Limitation of study design and analysis"</u>:

"It is important to note that we only tested here for linear relationships between our EEG and psychophysical measures. The literature on alpha-power predictors of performance in the visual domain has consistently shown evidence for a linear relationship between EEG and behaviour mostly with data binning methods

EJN European Journal of Neuroscience FENS Federation of European Neuroscience Societies

(Thut et al., 2006, Chaumon & Busch, 2014, Limbach & Corballis, 2016 , Iemi et al., 2017) suggesting that this is a reasonable starting point. However, previous studies have also found non-monotonic relationships between pre-stimulus oscillatory power and post-stimulus evoked neural activity and/or perception, primarily for tactile perception (Linkenkaer-Hansen et al., 2004, Zhang & Ding, 2010, Lange et al., 2012, Ai & Ro, 2014) but also with some evidence in the visual domain (Rajagovindan & Ding, 2011, Snyder et al., 2015). For instance, there is converging evidence that an intermediate level of pre-stimulus alpha power is optimal for detection of threshold tactile stimuli, with performance dropping off for trials with lowest and highest alpha power. The results of the current study do not rule out the possibility that such a relationship may also exist between oscillatory power and our psychophysical measures of interest. The jack-knife procedure allows to test for the nature of these relationships in more detail in future studies. "

Lesser concerns:

3) With respect to the potential influence of the phase of neural oscillations on perceptual performance, the authors might consider referring to the work of Ian Fiebelkorn (e.g., Fiebelkorn et al., 2011, J Neurosci; Fiebelkorn et al., 2013).

Thank you for this suggestion, we have now added citations of these relevant papers to the introduction (page 3) and the methods section (page 18).

4) There are two "Benwell et al., 2014" papers in the reference list, but there is no 'a' nor 'b' to enable me to tell which paper is meant when the authors cite "Benwell et al., (2014b)" on page 6.

Thank you for noticing this oversight, the relevant indexes have now been added to these references.

5) Since the authors find a new set of regression coefficients at each time-frequency-channel data point, the formula on page 13 may be more accurate if the beta terms are also indexed over time and frequency.

Thank you for this suggestion, we have changed the formula accordingly.

6) What do the authors mean by "condition labels" on page 14? Does this refer to which stimulus was shown, what the subjects' responses were, both, or something else? Why was the shuffling done only for a subset of the subjects on each iteration? How large was the subset? I would think leaving some of the subjects' data unshuffled would be overly conservative...

We apologise that we did not make this point clear and thank the reviewer for highlighting this. What was being shuffled on each iteration for a subset of the participants was the sign of the regression slope. Specifically, because we are performing a repeated measures one-sample t-test of regression slopes (versus 0) to test for a systematic direction of the regression slopes, in order to permute this data the sign of the regression slope was switched for a random subset of the participants. The number of participants included in the subset could vary on any given iteration as the choice of whether to switch the sign or not was selected randomly for each participant separately on each iteration. This is a standard method for conducting within subjects permutation reshuffling (i.e. it is the default method in FieldTrip). Based on the reviewer's comment, we have inserted the following on page 14 to try to clarify the issue:

"*Subsequently, this procedure was repeated across 2000 permutations to create surrogate data using 'ft_statistics_montecarlo' (Oostenveld et al, 2011). On each iteration, this function effectively switched the sign of the regression slope for a random subset of the participants. A one-sample t-test of regression slopes against zero was then performed at each data point. After clustering t-values across data points, the most extreme cluster-level t-score was retrieved in order to build a data driven null hypothesis distribution.*"

We hope that the process is now clear.

7) On page 15 the authors reference Figure 5, but I think from the context that they mean Figure 4 here.

Yes the reviewer is correct. Please also note that the figures have now been renumbered because of the addition of a stand-alone figure for the mediation model (based on a comment of reviewer 1) and all of the figures are now referred to correctly in the text.

8) What, exactly, was done for time-frequency transformation? The authors say on page 10 that they used a 0.5 s window and achieved 0.5 Hz frequency resolution. However if this was just short-time Fourier transformation then the frequency resolution should be 2 Hz for a 0.5 s window. Was this using the multitaper method? Something else (ft_freqanalysis is basically just a wrapper that supports several methods)?

We thank the reviewer for allowing us to clarify this point. We have now amended the section describing the time-frequency transformation on page 10 with the following:

"Fourier-based spectro-temporal decomposition of the artefact-removed single-trial data was performed using the ft_freqanalysis function (Oostenveld et al., 2011) with the 'mtmconvol' option. This implementation yields a complex-valued time-frequency plane for each trial. A temporal resolution was maintained by decomposing overlapping 0.5 sec segments of trial time series, consecutively shifted forward in time by 0.02 sec. Data segments were multiplied with a Hanning taper and then zero-padded to a length of 2 sec to achieve a frequency resolution of 0.5 Hz across the range of 1:30 Hz. The data were then re-epoched from -2:1 s relative to stimulus onset to exclude artifacts arising at the edges of transformed time series."

We hope that this eradicates any confusion regarding the TF transformation.

9) On page 9 it says 62 scalp electrodes were used, but on page 19 it says that 60 scalp electrodes were used. Please clarify.

60 scalp electrodes were used and we have amended the text accordingly on page 9.

10) Please expand the acronym "PFs" on first use (p. 18).

We have now expanded this on the first use.

11) When the authors state that effects "appeared to consist of two notable topographic patterns" (p.20, 21), how was this determination made? Just by eyeing the data, or was a more principled approach used somehow (e.g., microstate analysis)?

This decision was simply based on visual inspection of the topographic representations within the clusters, and the local minima in significance between the early and late negative "blobs" of the cluster. We now specify this in the text (page 22):

"The mostly post-stimulus cluster of negative power-PSE relationships was in the 6.5-16Hz frequency range and appeared to consist of two notable topographic patterns <u>(based on visual exploration of the effect)"</u>

Please note that we thought it useful to show the 2 topographies based on the local significance minima but we acknowledge that any conclusion derived from this observation is more indicative than conclusive. As a consequence, these two topographies are not a crucial part of our conclusions.

12) The Baron and Kenny procedure specifies that there should first be a significant relationship between X and Y to be mediated, but the total effect by the authors is reported as "t(10) = 1.9331, p = 0.0691" (p. 23), which is marginal. How valid is this mediation analysis if there is not clearly an effect to mediate?

Thank you for allowing us to address this relevant point. There are two reasons why we do not feel that the marginal significance of the total effect in this instance invalidates the mediation analysis:

1) *The trial order-PSE (time-on-task) effect (which the total effect represents) has been replicated several times previously in our own lab and across independent labs, as cited in the manuscript (Manly et al., 2005, Dufour et al., 2007, Benwell et al., 2013a, 2013b, most recently Veniero et al., 2017, Frontiers in Psychology). Indeed, in the current paper when we tested for this effect differently by separating the behavioural data into three blocks, we found a significant main effect of time-on-task on PSE (as reported in the 'Behavioural results' section on page 19). Hence, we are confident that there is a real effect to be mediated.*
2) *There is no consensus in the literature that statistical significance of the X-Y total effect is a pre-requisite for performing a valid mediation analysis. In fact, Zhao et al., (2009, Journal of Consumer Research, 37(2):197-206) state that "there need not be a significant X-Y link in a proper mediation analysis" (there is a section on this issue beginning on page 199 of their paper). Additionally, David Kenny himself more recently suggests that statistical significance of the X-Y relationship should not be adopted as a pre-requisite. The following is from his website(http://davidakenny.net/cm/mediate.htm):*
   "Note that the (mediation) steps are stated in terms of zero and nonzero coefficients, not in terms of statistical significance, as they were in Baron and Kenny (1986). Because trivially small coefficients can be statistically significant with large sample sizes and very large coefficients can be nonsignificant with small sample sizes, the steps should not be defined in terms of statistical significance."

13) The authors examined whether lateralization of alpha power was related to the response bias, but what about the same analysis for sensitivity?

The lateralization analysis was performed in order to facilitate comparisons to an extensive previous literature (Thut et al., 2006, Newman et al., 2013, Loughnane et al., 2015, Slagter et al., 2016, Newman et al., 2016), which report the relative lateralisation of alpha power to be linked to the degree of lateralised bias displayed on detection tasks. We felt it was important to test for this relationship with our lateralised bias measure (PSE) in order to inform the conclusions. However, we are not aware of evidence that the relative lateralisation of pre-stimulus alpha predicts discrimination sensitivity for tasks involving stimuli spanning both visual fields and had no theoretical basis to test for such a relationship.

In the new version of the manuscript, we now better justify in the relevant sections why we performed this additional analysis (see p. 17 and 26)

14) The legend to Figure 1 says that the next trial started as soon as the response was made, whereas the Methods section says that the next trial started 0.5 s later. Please clarify.

In response to this comment (and a comment of reviewer 1), we have now amended Figure 1 (and its legend) so that it now displays the trial procedure accurately.

15) I would like to have a better sense of the measures of interest. Would it be possible to include a distribution over jackknife repetitions of the bias and sensitivity estimates (and ITPC in the supplement)? I am wondering whether there is sufficient variation in sensitivity that an effect could be found. In other words, could one reason why the authors did not find pre-stimulus alpha-band power was related to sensitivity be because sensitivity didn't vary much during this task?

Please see our response to point 16 below.

16) More generally, the first data figure is already quite processed, showing only t-statistics for the distributions of regression coefficients: I would like to see some data in more "raw" forms, illustrating the intermediate analysis steps (e.g., a scatter plot comparing PSEs to alpha power, or something similar).

Thank you for this relevant point. Based on both points 15 and 16, we have now added a new supplementary materials section (the phase analysis has been moved into the main manuscript at the request of reviewer 1). We now show scatterplots of the relationships between EEG power and both jackknife PSE and curve width estimates collapsed across all participants (supplementary figure 1). We noted that the curve width measure is skewed. Hence, we also performed an additional median split analysis to compare the single trial jackknife approach with a more traditional binning (median split) approach. This additional analysis also allowed us to compare the sensitivity of the two methods. The results of this analysis are now displayed in supplementary figure 2. The results are broadly similar between the two approaches, though the jackknife seems to be more sensitive, at least for the PSE effects. We believe this is an important addition which allows the reader to get more of a feel for the data and the jackknife approach. We wish to thank the reviewer for this suggestion.

As already noted, we have reduced the emphasis on any interpretation of the discrimination sensitivity null result and have outlined alternative explanations in the new paragraph entitled: "Null result: No evidence for pre-stimulus power predicting visual sensitivity".

17) The axes labels for the inset panels in Figure 3 are too small to be legible.

Thank you for pointing this out, we have now increased the size of the axes labels for the inset panels in figure 3 and we hope they are now suitably legible.

18) The legend for Figure 3 states that significant electrodes are shown in white. Does that imply that every single electrode in the top of panel B is significant (including those that have a mean T-score of 0), and none of the electrodes in the bottom of panel B is significant? Everything (or very nearly) is white in the top row of the topographies, and everything is black in the bottom row. Is this right?

Yes the reviewer is correct. The topography shows the t-values from all electrodes averaged across all frequencies and time points associated with the cluster. The criterion for being highlighted as a significant electrode is simply that an electrode is included in the significant cluster at any time-frequency point at least once. Hence, for the pre-stim alpha cluster (Figure 4B), all electrodes were included in the cluster at some time-frequency point, though when averaged across all time-frequency-points, the t-value can be close to 0 (for instance if the electrode was only included in the cluster at very few data points).

The reason that the bottom row of surface Laplacian topographies have no electrodes highlighted is because we did not perform cluster-based statistical analysis on the Laplacian data. We employed this analysis to try to gain a more spatially specific estimate of the effects from the average reference analysis. We have now added the following to the figure 4 caption in order to clarify these points:

***"(B)** Topographical representations of the t-values associated with the pre-stimulus cluster (top row: electrodes that were significant at least once at any time-frequency point within the cluster are highlighted in white). Note that cluster-based permutation tests were not performed on the Surface Laplacian data (bottom row), rather this topography was calculated in order to provide a more topographically localised estimate of the effect."*

19) When the authors compute surface Laplacians, this is done on the data before time-frequency transformation, correct? It could be possible, albeit improper, to do the analysis in the other order…

The surface Laplacians were computed using the time-domain signals prior to time-frequency transformation. We have now stated this in the text (page 17).

20) In Figure 4, alpha band activity is given in units of uV/cm^2. However, the Methods section states that power values were expressed in decibels. Are the units right here? Even without the decibel transformation, the formula on page 10 would give squared microvolts (per square centimeter, for the Laplacian).

Thank you for allowing us to clarify this. For the lateralisation ROI analysis, we used Laplacian data and this was not db transformed. However, the reviewer is correct that the unit should be $(\mu V/cm^2)^2$

We have now amended this in Figure 5 (which was figure 4 in the original manuscript).

Reviewer: 3

Comments to the Author

In this study, Benwell et al examine the relationship between pre-stimulus alpha-band power and visuospatial bias and sensitivity in a landmark task. They support previous work in showing a relationship with bias but not sensitivity, and go further to show in a mediation analysis that this relationship could at least partially be driven by time-on-task effects. Both overall alpha and alpha lateralization were tested, and interestingly only the former correlated with perceptual bias.

The paper is written very well, with detailed and thorough methods and a comprehensive display of the results. My comments below relate to a couple of additional analyses that would be easy to do, but which the authors may have good reasons not to do and could clarify, and with going a little further in interpretation.

The subjects responded with their index versus middle finger to indicate their perception of the left versus right part of the line being shorter. Can the authors rule out that it could be a motor bias rather than a perceptual bias?

We thank the reviewer for raising this important issue. It is true that the PSE measure of subjective midpoint adopted here is potentially confounded by motor response bias, as participants always had to indicate which end of the line appeared 'shortest' of the two. This confound can be removed by alternating within participants trials in which they are requested to indicate the 'shortest' and 'longest' end of the line. Many previous landmark task studies employing either a single instruction (i.e., indicate the shortest) and/or separate instructions (i.e., alternating 'shortest' and 'longest' both within and across participants) have consistently shown a baseline leftward bias (pseudoneglect) in samples of healthy, young individuals so we

can be quite certain that the baseline leftward bias is unlikely to occur due to a motor response bias. It remains possible that shifts in PSE over time (time-on-task) may be confounded by a shift in or emergence of a response bias. However, the topographic representations of the pre-stimulus predictors of the PSE (including the deterministic effect highlighted by the mediation analysis) do not appear to support a motor origin for these effects. We have added a sentence to the discussion under a new subsection entitled "Limitations of study design and analysis" acknowledging this as a potential weakness of the study:

"One limitation of the current study is that participants always indicated which end of the line appeared to be 'shortest' with the same finger/response mapping. Hence we cannot rule out a potential influence of motor response bias on our subjective midpoint measures. Although the topographic representations of the identified EEG/spatial bias associations do not suggest a motor origin of the effects, future studies should alternate within and/or between participants the instruction to identify either the 'shortest' or 'longest' end of the line in order to eradicate the potential influence of response bias (Toraldo et al., 2004)."

Related to this, I was curious as to why the authors did not examine RT, since they mention a good deal of previous work showing effects of prestimulus alpha on RT. RT is presumably also something that could change with time on task, and may well be asymmetric for the two fingers used, which have a fixed mapping to the two sensory alternatives.

We agree with the reviewer that an analysis of pre-stimulus oscillatory predictors of landmark task RT and RT changes over time would be potentially of much interest. However, the reason that we did not examine this in the current study is because we judge the experiment to be sub-optimal for investigation of response speed. The idea behind the study was to investigate EEG predictors of separate parameters derived from the individually fitted psychometric functions (PSE and curve width) and we never intended to look at RT when designing the experiment. Hence, we did not instruct participants to provide a speeded response but rather to try to be as accurate as possible. In fact, because the following trial did not begin until the participant responded and we had tried to urge participants not to blink during the critical EEG periods of interest (i.e. prior to and during stimulus presentation), some participants developed a blink strategy by which they sometimes made their decision on a given trial but withheld their response until they had paused briefly to rest their eyes or blink.

In addition, most of the previous studies showing effects of prestimulus alpha on RT with regard to lateralised spatial biases have employed detection RT asymmetries as their measure of lateralised bias. However, here we employed the PSE but we predict that a landmark task RT asymmetry analysis, from an experiment suited to look at this, would give broadly similar results to our PSE analysis. This is an interesting open question for future research but unfortunately we do not think that the current study is suitable to investigate this.

Some of the interpretations are not fully clear in the paper as it currently stands. For example, I'm not sure that the mediation analysis has been interpreted fully and clearly – I see that it indicates that not all of the influence of time-on-task on PSE is direct, and that some of this is mediated through alpha, but what does it mean regarding the relationship between alpha and spatial bias itself? The paper is motivated by the need to distinguish "deterministic" versus "stochastic" sources of variability in alpha which may predict bias, but I'm still not sure after reading the paper how much of these two kinds of variability are in play in the data. It seems that in order to know whether it's not all time-on-task, i.e., that there is some additional variability in baseline alpha over and above the time on task trend, which further predicts bias, the authors would have to do a stepwise regression, i.e. once the direct influence of time on task is accounted for, does adding the

additional independent variable of alpha (perhaps most appropriately the residual variations in alpha after separately regressing out the effect of time on task) explain yet more variance in the behavioural bias?

We performed the suggested analysis for the peak data point of the pre-stimulus effect in order to answer the question as to whether we find evidence for any 'stochastic' predictor of spatial bias in the pre-stimulus effect on top of the time-on-task effect. The analysis confirmed that not all of the observed pre-stimulus power/PSE effect can be accounted for by the time-on-task induced shift in alpha power. We have added the following description of the results of the stepwise regression at the end of the Mediation analysis results section (page 25):

"In order to ascertain whether any additional 'stochastic' variability in pre-stimulus power, over and above the time-on-task trend, further predicts spatial bias, we performed an additional analysis. At the data point corresponding to the peak t-value of the jackknife analysis pre-stimulus cluster (14 Hz, -1.78 secs at electrode AF4), we retrieved the residual variations in power after regressing out the effect of trial order within each participant. We then collapsed the data across participants and performed a stepwise regression analysis (using the 'stepwisefit' function in Matlab) with z-scored (within-participants) jackknife PSE estimates as the response variable and trial order and the residual variations in power as the predictor variables. The analysis revealed a significant relationship between the residual variations in power and spatial bias (β = .036, t = 3.8730, p = .0001), even after accounting for the direct influence of time on task. Hence, we find evidence that both 'deterministic' and 'stochastic' sources of pre-stimulus EEG variability predict spatial bias, with the 'deterministic' aspect appearing to be restricted to a narrow, alpha-band specific time-frequency range (see Figure 5A)."

We now note the following in the discussion (page 33):

"In fact, we found evidence that both 'deterministic' and 'stochastic' sources of pre-stimulus oscillatory variability predict spatial bias on the landmark task."

We wish to thank the reviewer for this valuable suggestion. We believe this analysis provides important additional information and hence improves the manuscript.

Another matter that could use more discussion is the nature of this alpha relationship with bias. Specifically, what determines the direction of the bias? Why might greater alpha specifically relate to rightward as opposed to leftward bias in the current task? The authors suggest that an asymmetry in baseline excitability in neurons coding for one alternative versus the other might underlie the alpha effects, but this seems at odds with the fact that there was no spatially specific component of alpha that showed this relationship. That is, there is no evidence for alpha reflecting any kind of asymmetry in itself (end of page 28).

We agree that the interpretation in regards to bias in the former version of the manuscript was not fully clear. Based on the two main points of reviewer 2, we have toned down and/or eliminated those considerations on bias (versus sensitivity) that were aligned to a series of previous studies that linked pre-stimulus alpha power to decision criterion/bias (versus sensitivity) broadly looking at bias in the signal detection framework. If this was partially or fully the source of confusion, this is now eliminated.

We also reworked the sections where we discuss the link between pre-stimulus alpha, spatial bias and time-on-task more within an attention framework (and which is better aligned with the task we employed than the signal detection framework), to hopefully make our points clearer (please see discussion, first section). In short, we note that the surface Laplacian analysis hints at a right hemispheric (RH) locus, indicating that one explanation could be hemispheric lateralisation. We therefore interpret our finding of increased alpha over the right hemisphere over time leading to a rightward shift in bias (revealed by the mediation analysis) to be consistent with previous interpretations of right hemispheric disengagement (or depletion) to

lead to changes in attentional bias, away from the left, towards the right hemifield. We believe that a coherent explanation for the relationship between the time-on-task effect, alpha power and visuospatial attention/alertness networks is put forth in the discussion section entitled "Relation of pre-stimulus alpha-power over the right hemisphere is linearly related to spatial bias: Link to models of information flow and higher-order attention networks". Please see directly in the text for detail. .

Finally, the bias-predictive alpha activity comes and goes quite a long time before the stimulus onsets. Can the authors discuss why this might be? Most pre-stimulus effects tend to last right up until the stimulus actually occurs – is there some explanation for why this might not be the case here?

Thank you for highlighting this relevant point. We are not entirely sure why the effect comes and goes relatively early in this manner. However, we note that whilst the significant cluster offsets quite far from stimulus onset, the positive relationship between alpha power and PSE is actually quite sustained in time until closer to stimulus onset (see Figure 4A), though it is not reaching the threshold to survive cluster correction during this period.

One potential reason (though this is only speculation) as to why the effect might not be seen immediately prior to stimulus onset is that the timing of stimulus onset was always entirely predictable. Hence, alpha power may have decreased immediately prior to onset in anticipation of the stimulus and the effect may thereby have been destroyed by this anticipatory effect or alternatively may have been more difficult to pick up if it was there. In fact, if you look at figure 6A showing the raw left and right hemisphere ROI alpha power over the course of the trial, there is some evidence that this may indeed have been the case because the alpha power starts to markedly decrease just after 1 second prior to stimulus onset. Alternatively, and in addition, there may have been smearing of the alpha reduction post-stimulus into the pre-stimulus period making it more difficult to pick up any positive relationship between pre-stimulus power and behaviour in the late pre-stimulus window.

Please note that the early locus of the effect may also speak to the nature of the tonic/deterministic contribution uncovered by our mediation analysis which would not necessarily be expected to be as strongly stimulus locked as a purely phasic/stochastic effect.

Because the paper is already very long, we decided not to include these points, and hope the reviewer agrees

A general point on writing style: There is an overuse of parantheses throughout the paper. For clarity, I suggest writing additional full sentences rather than trying to cram too many points into each individual sentence. Also, the authors litter the paper with "see above" and "see below" statements, and so it's quite jarring to follow the narrative. I suggest choosing a sequence of ideas that can proceed linearly so that it isn't necessary to refer back and refer forward quite so much. Sometimes this can't be helped, but in cases like the second paragraph of the discussion, this is both inconvenient and maybe even misleading for the reader. "Our data suggest that posterior pre-stimulus alpha fluctuations can shape perception through influencing decision bias at higher order processing stages, potentially during readout of sensory evidence from sensory cortex, as discussed below" is actually quite a major claim and really needs to be justified immediately, not put off to several sections later. The claim itself could be postponed until the narrative is ready to address it fully.

Thank you for this valuable suggestion. We have now incorporated this in the manuscript by reducing unnecessary use of parentheses and jarring references to other sections of the manuscript throughout. We have tried to only introduce ideas/concepts at the point where they

are appropriately discussed when possible. We also avoided cross-referencing by reordering the presented discussion sections, e.g. begin with discussing all aspects of prestimulus predictors of spatial bias (the positive results) and moved the discussion of the negative result (sensitivity) towards the end. Accordingly, we have now removed the sentence quoted by the reviewer. We hope that the manuscript is now easier to read and understand.

Minor:

In the intro (p3), Kelly et al (2006) is cited in relation to alpha lateralization predicting RT, but I think the authors may mean Kelly et al (2009) in this instance.

We apologise for this oversight. We have now added the correct citation in the text on page 3.

Top of page 4 (intro): could the authors explain what they mean by “higher-order” in describing the task? I'm not sure what this might mean.

We thank the reviewer for bringing this to our attention, upon reflection we feel that this term is unnecessary and somewhat confusing within this sentence and so we have now removed it.

The paragraph starting “We here expand” is somewhat awkwardly worded and could use editing for clarification. The previous paragraph ends with the statement that bias, and not sensitivity, has been linked to prestimulus alpha; then this one starts talking about “two separate psychometric measures,” as if two new concepts are about to be introduced, but which turn out to be bias and sensitivity. I suggest first defining bias and sensitivity and how they are “separate,” then to cut straight to the new element of this study, which is to measure them in the landmark task.

Thank you for pointing this out. We have now changed the start of this paragraph in order to cut straight from the discussion of the perithreshold literature in the preceding paragraph to the rationale for our study:

“Here, we sought to investigate whether bias and/or sensitivity are influenced by pre-stimulus oscillatory activity during performance of a psychophysical task with suprathreshold stimuli.”

Methods:

Task: “The subsequent trial began 0.5 s after the response was made” - this seems to contradict the figure 1 legend. The numbers do add up, but the authors should state the timings consistently to avoid confusion.

We apologise for this oversight. Figure 1 and its caption have now been changed in order to accurately depict the trial procedure.

Do JKRi and si (small case 's') refer to the same thing? If so, use just one of them.

The reviewer is correct, JKRi and si indeed refer to the same thing. We have now replaced si with JKRi. Thank you for the careful reading of the manuscript.

2nd Editorial Decision 23 August 2017

Dear Dr. Benwell,

Your revised manuscript was re-evaluated by two external reviewers and ourselves. We are pleased to provisionally accept your paper for publication in EJN pending a few very minor amendments. First, and most importantly, Reviewer #2 points to some confusion in interpreting your equations and would like you to clear this up before we proceed to publication.

Please also attend to the following points:
1) Graphical abstract needed
2) Author contributions and data sharing statements are still needed

If you are able to respond fully to the points raised, we shall be pleased to receive a revision of your paper within 30 days.

Thank you for submitting your work to EJN.

Kind regards,
John Foxe &
Paul Bolam
co-Editors in Chief, EJN

Reviews:

Reviewer: 1 (Ulrich Pomper, University College London, UK)

Comments to the Author
The authors have thoroughly revised the manuscript, and answered all the points I have raised. I have no further questions or concerns, and consider the manuscript now suited for publication.

Reviewer: 2 (Adam Snyder, University of Pittsburgh, USA)

Comments to the Author
The authors have addressed my prior concerns. However, as I go over this again I'm tripped up by some of the description on pages 7-8. I think I know what the authors intend, but the current description is a bit confusing. The equations are not enumerated in the manuscript, so I will reproduce them here to ease discussion:

(1) B*X = log(mu/(1-mu))
(2) PSE = (logit_50 – A)/B
(3) Curve width = (logit_25 – logit_75)/2B

This is currently unclear because the variable 'B' is reused with two different meanings. In equation (1) it is the vector of coefficients for a line, whereas in (2) and (3) it seems to refer to the slope parameter of a logistic function.

When elaborating on equation (2) that "...A and B are the coefficients returned by the logit fit." Based on the description provided, the authors are not fitting a logistic function to the data directly. Rather, they are applying the logit transform to the data and then fitting a line to those transformed values (which is in a sense equivalent, but the current description adds to confusion about what the coefficients mean).

By working backwards from equations (2) and (3), I determined that the coefficients in those equations determine a logistic function defined as

(4) mu = 1/(1+exp(-(Bx+A))

This definition should be made explicit in the manuscript. It would be helpful to not re-use B with two meanings. For my own ease of description, let me define 'B' from equation (1) as the column vector B = [m ; b]. Since the authors didn't directly fit the logistic function to the data, I believe they found A and B (from eq. 2 & 3) as B = m and A = -b/m, correct? It might help if this was also made explicit.

Note that logit_50 = 0, so equation (2) can be simplified to PSE = -A/B.

EJN European Journal of Neuroscience
FENS Federation of European Neuroscience Societies

Authors' Response 25 August 2017

We wish to thank the editor and the reviewers for their thorough and fair treatment of our manuscript. Based on the most recent comments, we have now uploaded a graphical abstract and added author contributions and data sharing information to the manuscript. Additionally, we have updated the equations associated with psychometric function descriptions on page 7 and 8 in line with the comments of reviewer 2. We wish to thank the reviewer for allowing us to clarify this. Changes to the text of the original submission have been underlined.
